# Elucidating ligand interactions and small-molecule activation in the pyrrolnitrin biosynthetic enzyme PrnB

**DOI:** 10.1016/j.jbc.2024.108123

**Published:** 2024-12-25

**Authors:** Bingnan Li, Remigio Usai, Jackson Campbell, Yifan Wang

**Affiliations:** Department of Chemistry, University of Georgia, Athens, Georgia, USA

**Keywords:** pyrrolnitrin biosynthesis, heme-dependent catalysis, spectroscopic characterization, crystal structure, heme-based oxidants, tryptophan oxidation

## Abstract

Pyrrolnitrin, a potent antifungal compound originally discovered in *Pseudomonas* strains, is biosynthesized through a secondary metabolic pathway involving four key enzymes. Central to this process is PrnB, a heme enzyme that catalyzes the complex transformation of 7-Cl-*L*-tryptophan. Despite its structural similarity to indoleamine 2,3-dioxygenase and tryptophan 2,3-dioxygenase and its classification within the histidine-ligated heme-dependent aromatic oxygenase superfamily, PrnB has remained relatively unexplored due to the challenges in reconstituting its *in vitro* activity. In this work, we investigated the interactions of PrnB from different strains with its substrates, substrate analogs, and small molecules using various biophysical and biochemical techniques. Our spectroscopic data reveal that the substrate amino group directly coordinates with the heme in both oxidized and reduced enzyme forms. This binding conformation was further confirmed by X-ray crystallography of enzyme-ligand binary complexes. The amine ligation inhibits H_2_O_2_ and CN^-^ from interacting with the ferric heme but does not notably impact ^•^NO binding or O_2_ activation by the ferrous heme. Stopped-flow spectroscopy showed the formation of heme-based oxidants similar to those reported in indoleamine 2,3-dioxygenase and tryptophan 2,3-dioxygenase when PrnB was exposed to H_2_O_2_ or O_2_. However, these intermediates lacked catalytic activity, and PrnB was inactive when coupled with common redox systems under various conditions. This suggests that PrnB operates through a catalytic mechanism distinct from other heme-dependent aromatic oxygenases and most heme enzymes. Our study provides new insights into ligand binding and small-molecule activation mechanisms of PrnB, highlighting its unique functionality and distinguishing it from existing paradigms in heme catalysis.

Pyrrolnitrin is a microbial pyrrole halometabolite with significant antimicrobial applications in the agricultural, pharmaceutical, and biotechnological industries (1). First isolated from *Pseudomonas pyrrocinia* in 1964, it has since been used as an active component of the antifungal drug Pyroace (2). The pyrrolnitrin biosynthetic pathway comprises four enzymatic steps, each involving a conserved enzyme (Fig. 1) (3). Starting from *L*-tryptophan (Trp), the biosynthesis begins with the 7-chlorination of the indole ring by PrnA. PrnB then catalyzes a ring rearrangement to form monodechloroaminopyrrolnitrin, followed by the second chlorination and final amine-oxidation by PrnC and PrnD, respectively, to produce the final metabolite. Introduction of the *prnA*-*prnD* gene cluster to *Escherichia coli* resulted in pyrrolnitrin production, demonstrating the sufficiency and necessity of the four genes for *in vivo* biosynthesis (4, 5, 6). The biosynthetic gene cluster is also conserved in other pyrrolnitrin-producing bacteria (7). This pathway has gathered much attention since its discovery, with all four enzymes characterized at the molecular level. PrnA and PrnC are flavin-dependent halogenases (8, 9), PrnB is a histidine-ligated heme enzyme (3, 10), and PrnD is a Rieske *N*-oxygenase (11, 12).

**Figure 1:**
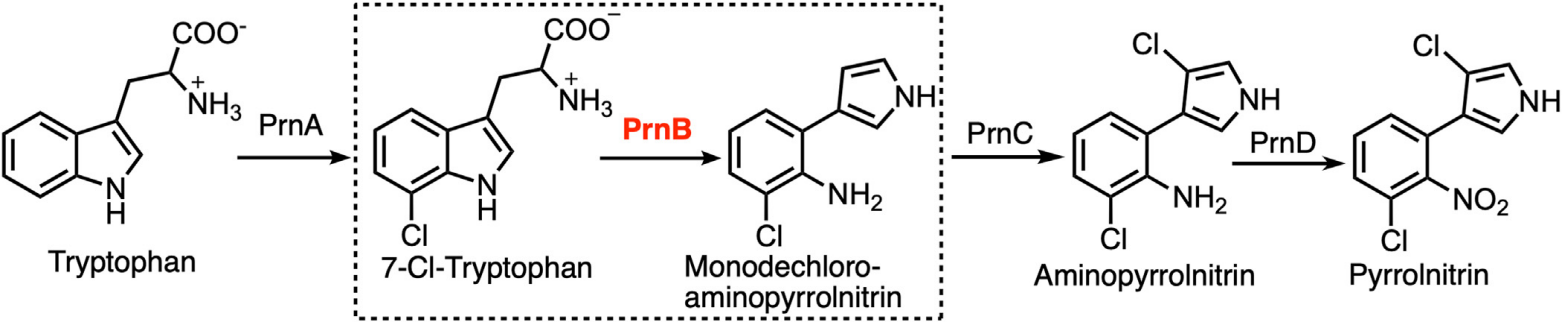
**The pyrrolnitrin biosynthetic pathway consists of four enzymatic steps.** PrnB catalyzes the biotransformation of 7-Cl-Trp in the second step.

PrnB catalyzes a significant biotransformation among the four enzymatic steps, constructing the skeletal structure of pyrrolnitrin. While heme enzymes are known for their abilities to catalyze redox reactions, the reaction catalyzed by PrnB is unique and complex, involving C-N bond cleavage, C-N bond formation, C-H bond activation, and decarboxylation. This transformation is a substrate oxidation process that is biochemically interesting, since it relies on a His-ligated heme cofactor rather than the more common Cys-ligated heme in redox reactions. Although PrnB has been known for almost three decades, its *in vitro* activity has only been reconstituted with fresh crude cellular extract from *Pseudomonas*. Incubating the substrate, 7-chloro-*L*-tryptophan (7-Cl-Trp), with the isolated enzyme did not yield any detectable product, monodechloroaminopyrrolnitrin (3, 10). Despite its unique enzymatic function, PrnB has been relatively unexplored due to the absence of *in vitro* activity. A better molecular understanding is needed to elucidate PrnB’s activity and further its mechanistic study.

Naismith *et al.*, established that PrnB belongs to the indoleamine 2,3-dioxygenase (IDO) and tryptophan 2,3-dioxygenase (TDO) superfamily due to its high structural similarity (3, 13). IDO and TDO oxidize Trp to N′-formylkynurenine, the initial and rate-limiting step in the kynurenine pathway that is highly relevant to pathological conditions and tumor resistance (14). Hence, IDO and TDO are considered important drug targets. Later bioinformatic studies and biochemical characterization of some related oxygenases have expanded this small enzyme family into a histidine-ligated heme-dependent aromatic oxygenase (HDAO) superfamily (15). These oxygenases are primarily found in the biosynthesis of natural products with important antimicrobial and antitumor activities, including TyrH in lincomycin and anthramycin biosynthesis (16, 17), SfmD in saframycin biosynthesis (18, 19), and MarE in maremycin biosynthesis (20). PrnB and IDO are among the most closely related members of the HDAO superfamily (15). However, they share less than 20% protein sequence identity and catalyze very different reactions involving indole transformation. This discrepancy may be partially attributed to differences in the distal heme pockets and substrate-binding conformations, but the precise molecular basis for these divergent outcomes remains unclear. It is also uncertain whether the catalytic mechanism of PrnB aligns with what we have learned from IDO/TDO or other HDAOs. An investigation of its heme environment and interactions with substrates and small molecules is essential to bridge this knowledge gap and help us appreciate the diversity within HDAOs.

PrnB can also accept *L*- and *D*-Trp and *D*-7-Cl-Trp as alternative substrates, though the *L*-forms are considered the natural substrates due to their bioavailability (3, 21). In the previously reported complex structures of PrnB (3), the indole rings of 7-Cl-Trp and Trp bind to PrnB in distinct conformations, while the amino groups of both ligands directly coordinate with the heme. It is unclear how these different binding conformations of 7-Cl-Trp and Trp lead to similar catalytic outcomes. Additionally, the direct amine ligation to the heme center is unexpected, as the sixth coordination site in heme enzymes is typically reserved for oxidants such as O_2_ or H_2_O_2_. It is crucial to understand whether this substrate-binding mode is conserved in solution and whether it is common among other PrnB homologs, as well as whether such binary complexes are susceptible to small-molecule binding and activation.

In this work, we report the ultraviolet-visible absorption, electron paramagnetic resonance (EPR), and resonance-enhanced Raman (rR) spectroscopic characterization of PrnB and its complexes with 7-Cl-Trp and Trp at different oxidation states. Two substrate analogs, tryptamine (TAM) and 3-indolepropionic acid (IDPA), are also tested to understand the roles of the amino acid moiety in substrate binding. The interactions between PrnB and small molecules, including CN^−^, H_2_O_2_, ^•^NO, and O_2_, are investigated in the presence and absence of the substrates. Transient kinetics obtained by stopped-flow absorption spectroscopy enables comparison with the catalytic mechanisms of HDAOs. The results provide molecular insights into how the heme center of PrnB interacts with ligands and potential oxidants, revealing a novel substrate binding mode in heme enzymes and offering new perspectives on the catalytic mechanisms of PrnB.

## Results

### Preparation of PrnB homolog proteins

Previous studies of PrnB encountered difficulties in obtaining soluble proteins using *E. coli* expression systems. Hence, *Pseudomonas fluorescens* was used as the expression host, and a Cys-to-Ser triply mutated variant was developed to enhance the protein stability for molecular studies (3). In this work, we used a PrnB homolog from *Pseudomonas sp.* ADAK18 (PsPrnB) for spectroscopic studies and a homolog from *Flavobacteriales bacterium* (FbPrnB) for crystallization studies. These homologs share 87% and 33% protein sequence identity with the *P*. *fluorescens* PrnB (PfPrnB), respectively (Fig. S1). Key residues critical for heme, substrate, and small molecule binding are strictly conserved between these PrnB homologs. FbPrnB was readily expressed as a soluble protein in *E. coli*. With the aid of chaperone proteins GroEL and GroES, PsPrnB was also successfully expressed in *E. coli*. (Fig. S2). Both proteins exhibited high heme occupancy (>70%).

### UV-visible absorption spectroscopic characterization of ligand binding affinities

**Figure 2:**
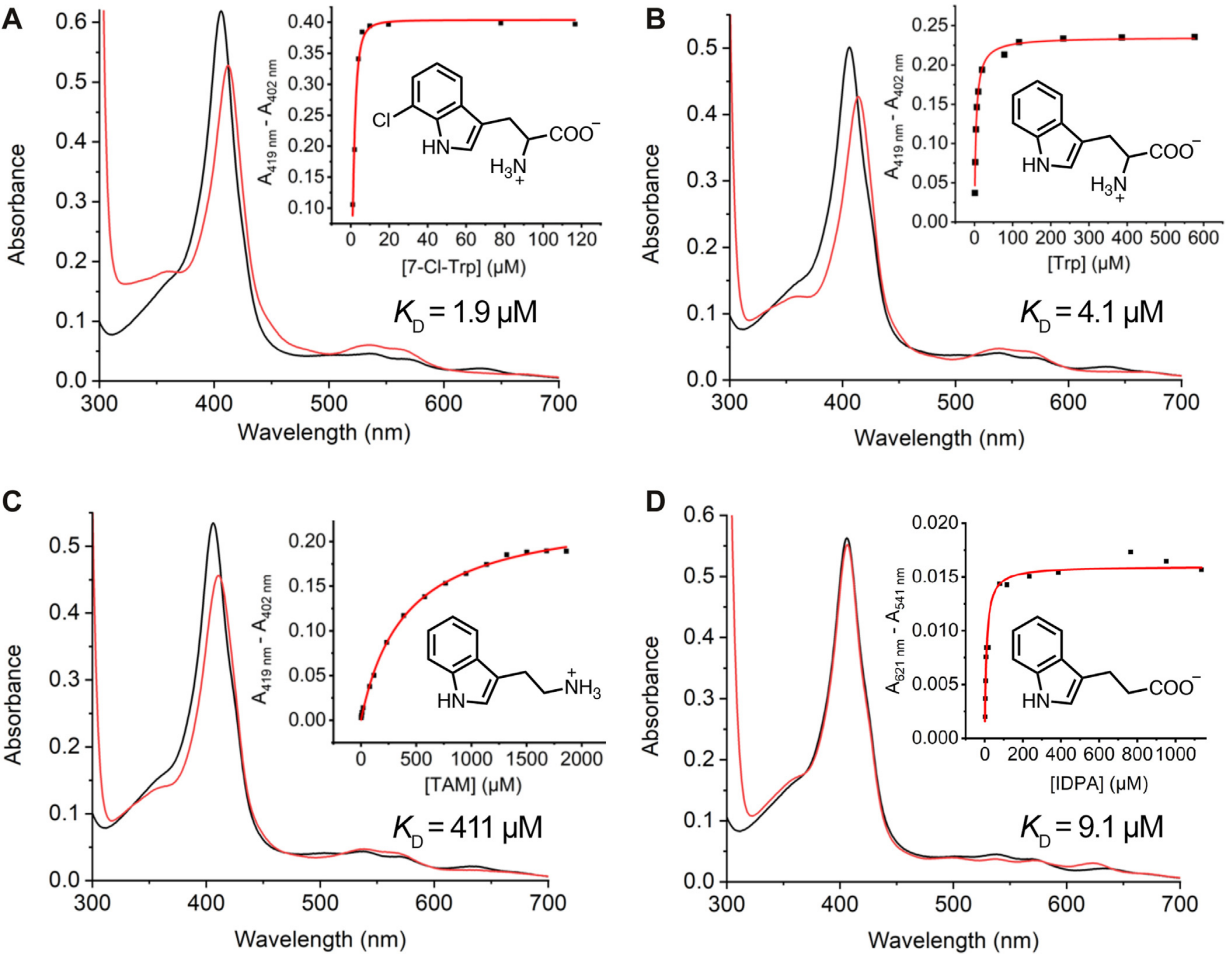
**UV-visible absorption spectral characterization of ferric PsPrnB.** Enzyme alone (*black*) and enzyme in complex with (*red*) (*A*)1 mM 7-Cl-Trp, (*B*) 1 mM Trp, (*C*) 1 mM TAM, and (*D*) 1 mM IDPA. (insets) The difference in absorbance (peak minus trough) as a function of ligand concentrations. Experimental data (*black dots*) are fitted to a hyperbola equation (*red lines*) to determine the *K*_D_ values. Samples were prepared with a protein concentration of 10 μM.

The resting state of PsPrnB exhibited a Soret band at 406 nm as well as Q and charge transfer bands at 536, 568, and 636 nm. Addition of 1 mM 7-Cl-Trp resulted in a 6-nm redshift of the Soret band, concurrent with changes in the Q bands to 535 and 563 nm and the disappearance of the 636-nm charge transfer band (Fig. 2*A*). The spectral features are consistent with the reported PfPrnB (3), indicating similar heme binding sites in these PrnB homologs. Substrate-induced spectral changes were also observed in the reduced form, where the Soret band at 426 nm became more pronounced, accompanied by shifts in Q bands upon substrate binding (Fig. S3*A* and Table S1). These unique spectroscopic changes, which are not observed in other HDAO enzymes, suggest a distinct substrate-binding mode in PrnB.

The spectrum of the Trp-bound ferric enzyme-substrate (ES) complex is akin to that of 7-Cl-Trp with slight differences in the wavelength maxima (Fig. 2*B* and Table S1), and the spectra of Fe^II^-ES complexes are identical (Fig. S3*B*). Two substrate analogs, TAM and IDPA, were also tested to investigate if the spectroscopic changes are related to the ligation of the amino acid moiety. Compared to Trp, TAM does not contain the carboxylate group, and IDPA lacks the amino group. In the oxidized form, the TAM-bound enzyme exhibited similar spectral features to the substrate-bound protein, whereas the IDPA-bound enzyme showed a distinct spectrum (Fig. 2, *C* and *D*). However, in the ferrous state, the spectral features of the TAM- and IDPA-bound enzymes were similar, and both spectra were distinct from the substrate-bound complexes (Fig. S3, *C* and *D*). The results suggest that the amine has a large impact on the heme electronic structure. Upon reduction of the heme, TAM changed its binding to a conformation more similar to IDPA. The carboxylate group seems to be critical for the ligands to adopt a substrate-like binding conformation in the ferrous state.

These spectral changes allowed us to measure the binding affinities (*K*_D_) of each ligand (Fig. 2, insets). Both 7-Cl-Trp and Trp bound tightly, with *K*_D_ values of 1.9 μM and 4.1 μM, respectively, which are comparable to previously reported values for PfPrnB (3, 10). Due to the strong binding, the measured *K*_D_ for 7-Cl-Trp may represent an upper-limit determination, and the actual value may be lower than what is reported. IDPA also bound tightly to the enzyme (*K*_D_ = 9.1 μM) albeit in a different manner, whereas TAM showed a dramatically decreased binding affinity (*K*_D_ = 411 μM). The carboxylate groups on 7-Cl-Trp and Trp likely decrease the amine p*K*_a_ due to an inductive effect, facilitating substrate coordination through a neutral amine. The *K*_D_ values indicate that the carboxylate group is important for substrate recognition; the amino group contributes less to substrate stabilization in the active site but significantly affects the heme center. Our spectroscopic data show that the interaction between amine and iron is relatively weak and susceptible to heme redox changes.

### EPR and rR characterization of heme interactions with substrates and analogs

**Figure 3:**
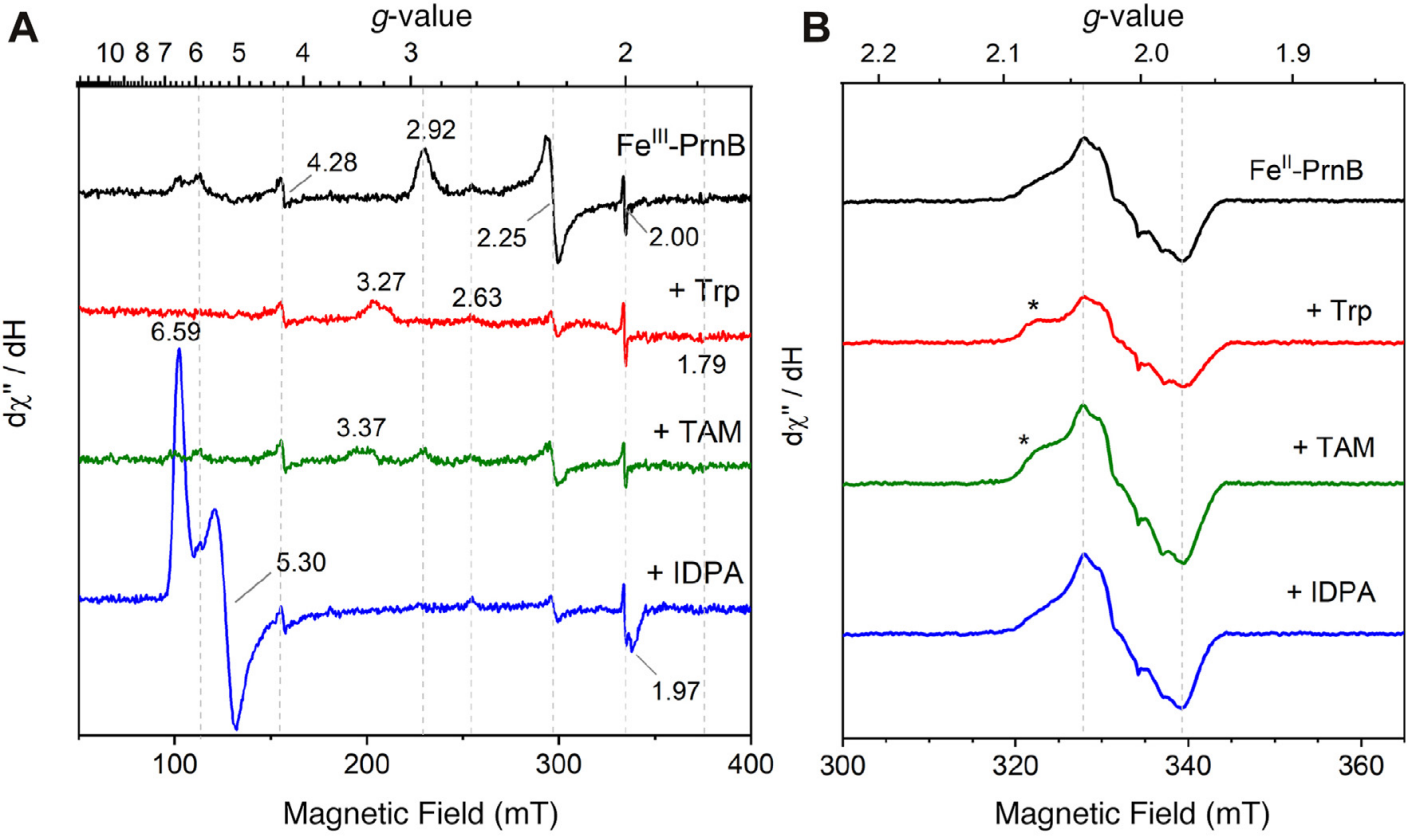
**X-band EPR spectra of ferric PsPrnB and nitrosyl complexes.***A*, from *top* to *bottom*: ferric enzyme alone (*black*), enzyme with 1 mM Trp (*red*), 1 mM TAM (*green*), and 1 mM IDPA (*blue*). The spectra were collected at 30 K and 1.0 mW microwave power. *B*, from *top* to *bottom*: nitrosyl complexes generated by ferrous enzyme alone (*black*), enzyme with 1 mM Trp (*red*), 1 mM TAM (*green*), and 1 mM IDPA (*blue*). The spectra were collected at 30 K and 0.05 mW microwave power. Additional features at *g* = 2.07 (Trp) and 2.08 (TAM) (marked by *asterisk*) were observed in Trp- and TAM-bound nitrosyl complexes. Samples were prepared with a protein concentration of 73 μM.

**Figure 4:**
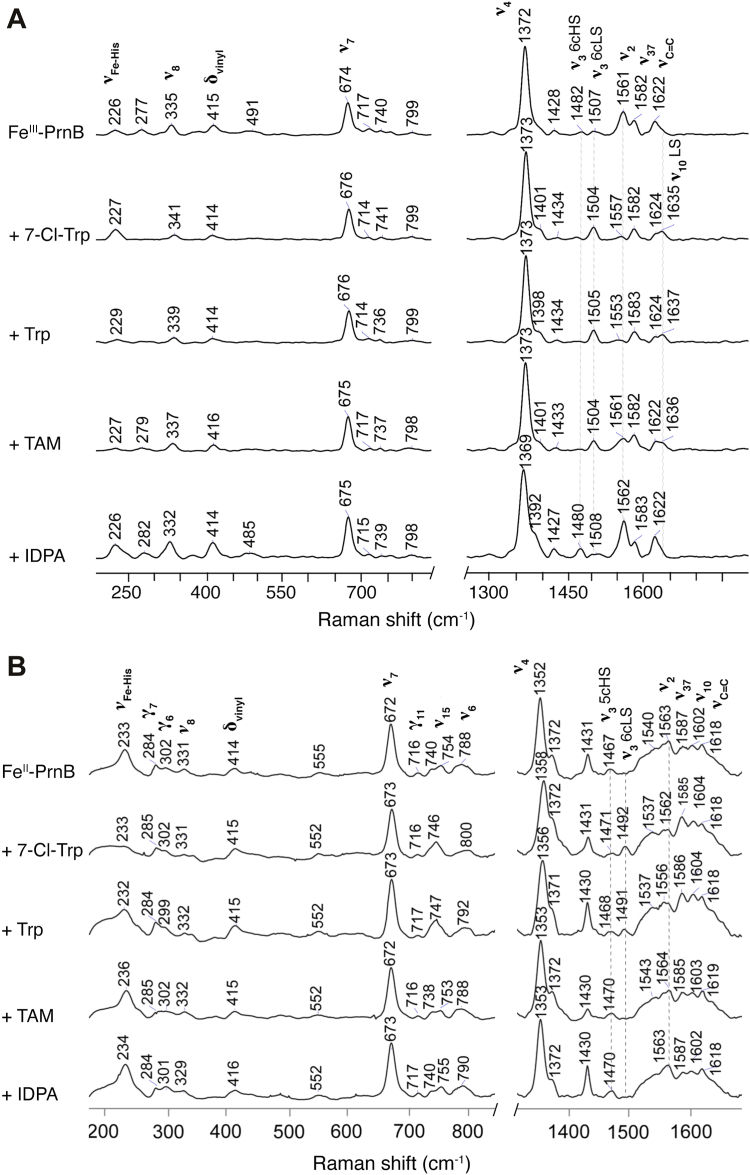
**Resonance Raman spectra of (*A*) ferric and (*B*) ferrous PsPrnB in the presence and absence of ligands.** From *top* to *bottom*: enzyme alone, enzyme with 1 mM 7-Cl-Trp, Trp, TAM, and IDPA. Low-frequency region (*left*) and high-frequency region (*right*) are shown. Ferric and ferrous samples were measured with an excitation line at 405 and 442 nm, respectively, at room temperature. Samples were prepared with a protein concentration of 135 μM.

Continuous-wave X-band EPR and rR spectroscopies were used to further characterize interactions between the substrates, analogs, and heme center. The majority of the Fe^III^-PsPrnB was in a low-spin state at *g*_z_ = 2.92, *g*_y_ = 2.25, and *g*_x_ = 1.79, with only a small fraction appearing as high-spin heme in the *g* = 6 region (Fig. 3*A* and Fig. S4). Trp binding to the enzyme resulted in the depletion of both the low- and high-spin signals, and a highly rhombic, low-spin signal was observed with a *g*_max_ value of 3.27. Similar highly anisotropic low-spin (HALS) signals have been reported in *bis*-ligated heme enzymes (22). The HALS signal was further analyzed at 10 K and simulated with *g*-values of 3.27, 1.98, and 1.19 (Fig. S5). Ligand-field correlation analysis suggests the rhombic (*V*/ξ) and axial (Δ/ξ) ligand-field parameters of 1.18 and 3.78, respectively, and a rhombic to axial ratio *V*/Δ of 0.31, values indicative of an environment more similar to HALS species involving a proximal neutral histidine (22). Microwave power saturation experiments show a *P*_1/2_ of 11.2 mW and a line shape factor *b* of 1.45, which distinguishes this HALS species from those with His-Met and His-Tyr coordination (23, 24). The disappearance of the original high- and low-spin signals was not due to the reduction of heme, since our rR spectral data indicate the heme remained in the ferric oxidation state (Fig. 4). Adding TAM to the enzyme resulted in partial conversion of the resting state to a low-spin species at *g* = 3.37, similar to the Trp-bound sample. Based on our UV-visible data and the reported crystal structures, we propose that the newly formed low-spin species is a six-coordinate heme with an amine axial ligand. In the presence of IDPA, a high-spin species (*S* = 5/2) was detected at *g*_z_ = 6.59, *g*_y_ = 5.30, and *g*_x_ = 1.97 resulting from the destabilization of the axial solvent molecule. Such a high-spin species is typical of HDAOs in the resting state (25, 26), and the *g*-values suggest an *E*/*D* value of approximately 0.022 based on the *S* = 5/2 rhombogram. Some minor signals were present in all samples. They are attributed to adventitious iron (*g* = 4.28), an inert low-spin heme species (*g* = 2.63 and 2.25), and a radical-like signal (*g* = 2.00) that are possibly due to freezing artifacts.

The reduced heme is EPR silent. Therefore, we used rR spectroscopy to probe the spin state and coordination environment of ferric and ferrous PsPrnB in solution. The assignments of the vibrational modes are based on those from other His-ligated heme proteins, including IDO, TDO, and hemoglobins (27, 28, 29, 30, 31, 32, 33, 34). The high-frequency region of the ligand-free PsPrnB displayed features characteristic of the ferric state, with a *ν*_4_ oxidation state marker at 1372 cm^−1^ (Fig. 4*A*). The *ν*_3_ spin state marker bands indicated the presence of a mixture of six-coordinate high-spin (6cHS) (*ν*_3_ at 1482 cm^−1^) and six-coordinate low-spin (6cLS) (*ν*_3_ at 1507 cm^−1^) species, consistent with our EPR measurements (Fig. 3) (28, 35). The complexes formed by 7-Cl-Trp, Trp, and TAM showed a predominant 6cLS species with *ν*_3_ at 1504 or 1505 cm^−1^. The *ν*_2_ band at 1561 cm^−1^, associated with high-spin heme, disappeared in the 7-Cl-Trp and Trp complexes and partially disappeared in the TAM complex, but was prominent in the IDPA complex. The *v*_10_ mode around 1635 cm^−1^, indicative of low-spin heme, was only seen in the 7-Cl-Trp, Trp, and TAM complexes. The IDPA-bound complex exhibited a six-coordinate high-spin signal (*ν*_3_ at 1480 cm^−1^) as the major component, with marginal low-spin species remaining, which is also consistent with the EPR spectra.

In the low-frequency region, the 226 ∼ 229 cm^−1^ bands are assigned to the proximal Fe-His stretching mode (*ν*_Fe-His_). The 335 cm^−1^ band corresponds to the Fe-porphyrin nitrogen stretching mode (*ν*_8_) with the vinyl bending character (29). The vinyl bending mode (*δ*_vinyl_) appeared at 414 ∼ 416 cm^−1^, indicating the vinyl groups are in plane (no activation of out of plane vinyl modes). We also noted that propionates are out of plane due to absence of an in-plane bending mode (*δ*_propionate_) around 387 cm^−1^. These observations are consistent with the orientations of the heme peripheral substituents as depicted in our crystal structures (see below). Ligand binding did not change the conformation of the peripheral substitutions, as these frequencies were highly conserved in all samples.

The rR spectra of Fe^II^-PsPrnB are shown in Fig. 4*B*. The enzyme exhibited a *ν*_4_ signal at 1352 cm^-1^. The binding of 7-Cl-Trp and Trp upshifted the band to 1358 and 1356 cm^−1^, respectively, but TAM- and IDPA-bound samples had no significant changes. In all cases, the oxidation state marker bands indicate the presence of a ferrous heme, which is sensitive to ligand binding. A minor peak at 1372 cm^−1^ was also observed, which does not correspond to the oxidized heme, as excess dithionite was used to reduce the heme center. Additionally, other spectroscopic methods did not detect the presence of ferric heme before or after the sample measurements. The origin of the 1372 cm^−1^ band is unclear, but the feature has also been observed in cyanobacterial hemoglobin (33). Fe^II^-PsPrnB, as well as TAM- and IDPA-bound complexes, contained a five-coordinate high-spin (5cHS) species with the spin state marker band *ν*_3_ at 1467 or 1470 cm^−1^. The *ν*_3_ band for the 7-Cl-Trp-bound complex indicated the presence of a mixture with the major component being the 6cLS (*ν*_3_ at 1492 cm^−1^) and a minor 5cHS species (*ν*_3_ at 1471 cm^−1^). The Trp-bound complex exhibited a similar mixture (*ν*_3_ at 1468 and 1491 cm^−1^). Collectively, we think that the amine of TAM is no longer an axial ligand upon heme reduction, while the amine of substrates remains as a ligand of the ferrous heme, possibly due to steric hindrance of the carboxylate group.

In the low-frequency region, the *ν*_Fe-His_ bands were observed at 233 cm^−1^, which are comparable to those in IDO (236 cm^−1^) and TDO (229 cm^−1^) (28, 29). The *ν*_Fe-His_ value falls into Class III Fe-His stretching, suggesting the axial His is likely in the imidazolate form and forms a strong coordination bond with iron (30, 31). The *ν*_Fe-His_ remained almost unchanged when bound to 7-Cl-Trp and Trp, but it slightly upshifted in the complexes with TAM and IDPA. However, 7-Cl-Trp and Trp binding caused noticeable disappearance of the *ν*_Fe-His_ band. This phenomenon has been observed before and indicates the formation of 6cLS ferrous heme (36). The *δ*_vinyl_ bands occurring at 414 to 416 cm^−1^ indicate in-plane vinyl, consistent with ferric samples. The activation of out-of-plane mode for the propionates was evidenced by *γ*_7_ bands around 284 cm^−1^. Overall, the reduction of the iron center enhanced the bond strength of Fe-His but did not change the conformation of the heme peripheral substitutions.

### Crystal structures of PrnB in complex with substrates and ligands

Although PsPrnB could be crystallized, the crystals did not yield high-quality diffraction data. As a result, we turned to FbPrnB for crystallization studies. The heme center and its interactions with Trp in FbPrnB closely resemble those of PsPrnB, as confirmed by similar UV-visible and rR spectra (Fig. S6). The crystal structure revealed two protein molecules in the asymmetric unit, while gel filtration indicated that the protein behaves as a monomer in solution, a finding supported by PDBePISA analysis (37). The majority of the protein was resolved in the crystal structure, with only the *N*-terminal His-tag and a few *C*-terminal residues remaining unresolved. These structures feature an *N*-terminal cap domain and a core helical domain typical of the IDO fold. The overall structures align closely with the previously reported PfPrnB, showing a RMSD of 1.12 Å for 289 C_α_ atoms.

**Figure 5:**
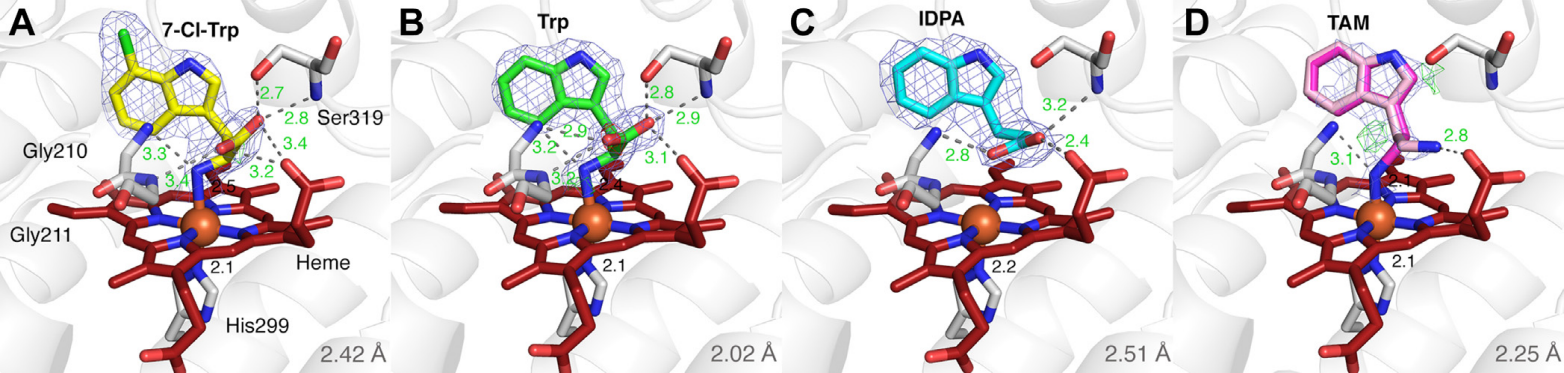
**Crystal structures of FbPrnB in complex with substrates and analogs.** Binary complexes with (*A*) 7-Cl-Trp, (*B*) Trp, (*C*) IDPA, and (*D*) TAM. The 2*F*_o_–*F*_c_ difference maps (*blue*) for the ligands are contoured at 1.5 σ. The *F*_o_–*F*_c_ difference maps (*green* and *red*) for the ligands are contoured at ±3.0 σ. Ligand interactions are represented by *gray dashed lines*, with distances (Å) labeled in *green*. Coordination bond distances (Å) are labeled in *black*. Color coding of atoms: C (protein)–*white*; C (heme)–*deep red*; N–*blue*; O–*red*; C (7-Cl-Trp)–*yellow*; C (Trp)–*green*; C (IDPA)–*cyan*; C (TAM, conformation 1)–*magenta*; C (TAM, conformation 2)–*pink*. Chain A was selected to show. The resolutions (Å) of each structure are indicated in the *bottom right corner*.

The active sites of all four binary complexes show electron densities for the ligands (Fig. 5), allowing us to clearly visualize their binding modes. In all cases, the indole rings of the ligands are oriented perpendicularly to the heme, but the positioning of the amino acid groups varies. Consistent with our spectroscopic data, the binding conformations of 7-Cl-Trp and Trp are nearly identical (Fig. S7*A*), with the amino group directly coordinating to the iron center, while the carboxylate group is stabilized by interactions with Gly210, Gly211, Ser319, and a heme propionate group. There are two notable differences when comparing our FbPrnB structures to those of PfPrnB (3). First, a previously disordered loop in PfPrnB (residues 323–331) becomes ordered in FbPrnB structures (residues 312–323), where it plays a role in stabilizing the substrate carboxylate group through Ser319 (Fig. S8). This loop closes over the substrate-binding site in the presence of ligands, likely aiding the enzyme in positioning the substrate correctly for efficient catalysis. Another major difference lies in the binding poses of Trp. While the amino acid moieties superimpose well between the Trp-bound structures, the indole rings adopt distinct orientations (Fig. S7*B*). The difference is primarily attributed to the substitution of Val117 in PfPrnB with Tyr104 in FbPrnB, which reduces the size of the active site pocket and restricts the indole positioning in FbPrnB (Fig. S8). In contrast, the binding poses of 7-Cl-Trp are similar between the two homologs (Fig. S7*C*), likely due to the 7-chlorine substitution, which locks the indole ring into a similar orientation in both structures.

The structure of the IDPA-bound complex aligns well with our spectroscopic data, confirming that IDPA does not directly interact with the iron center. The indole ring of IDPA adopts a similar orientation to that to Trp (Fig. S7*D*), but the propionic acid group rotates toward Gly210, forming a tighter interaction with the heme propionate (Fig. 5*C*). This aligns with our *K*_D_ measurements, which show that the removal of the carboxylate group significantly weakens ligand binding.

The TAM-bound structure yielded particularly intriguing results. Due to its weak binding (with a *K*_D_ 100-fold higher than that of Trp), the electron densities of TAM are not as strong as those of other ligands, and the occupancy was refined to 75% and 90% for Chain A and Chain B, respectively. The amine tail is flexible and adopts two distinct conformations in Chain A (Fig. 5*D*), with one remaining in coordination with the heme (40% occupancy), while the other rotates away to interact with the heme propionate group (35% occupancy). rR spectroscopic data revealed that the TAM-bound complex transitions from a 6cLS to a 5cHS state upon heme reduction. The reduction likely occurred during synchrotron data collection, resulting in the observed amine-off conformation. Interestingly, Chain B showed a different binding conformation in which the amine remained as a ligand, but the indole ring was shifted by 1.3 Å (Fig. S7*E*). The presence of multiple conformations in this binary complex suggests that TAM exhibits great lability due to the absence of a stabilizing carboxylate group and that the amine coordination is redox-sensitive.

### Interactions of ferric PrnB with cyanide and hydrogen peroxide

HDAOs readily bind and react with small molecules in the ferric form (19, 25, 32, 38, 39). Our spectroscopic and structural data show that the substrate amine binds to the ferric heme of PrnB, distinct from other HDAOs. A natural question is whether the substrate ligation interferes with small-molecule binding and activation.

**Figure 6:**
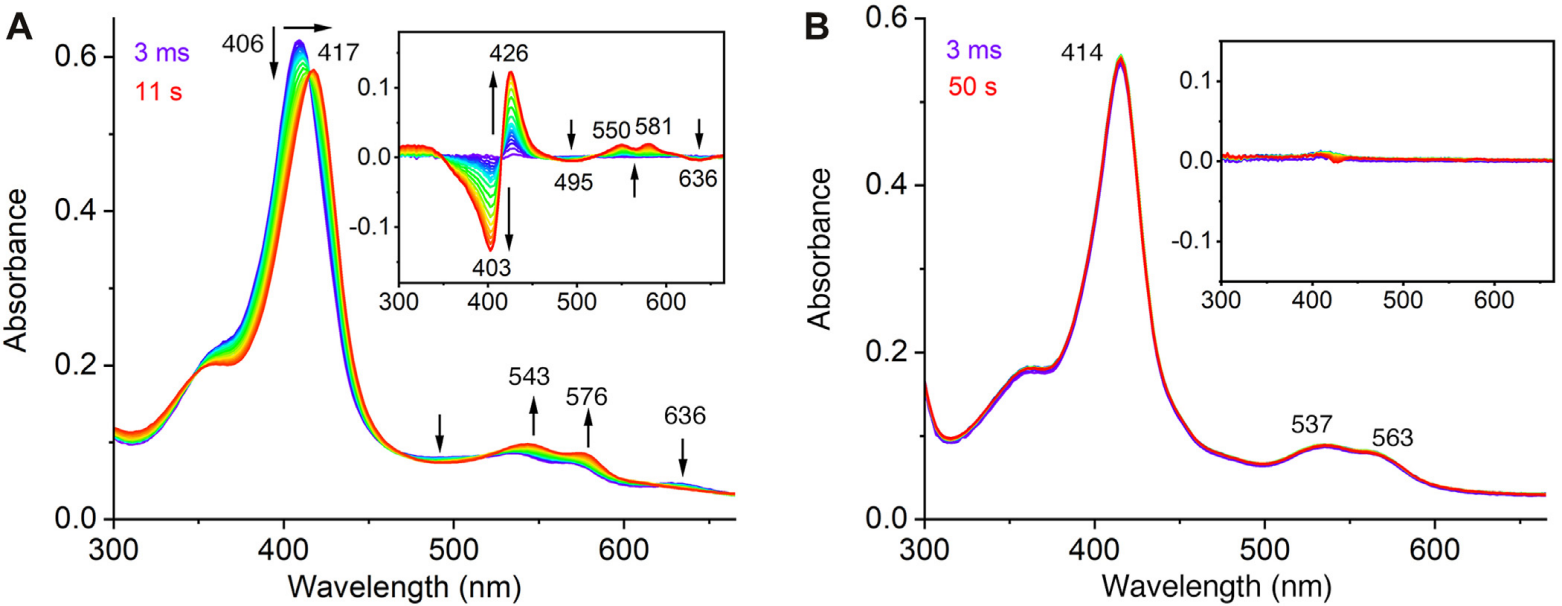
**Stopped-flow photodiode array spectra of ferric PsPrnB mixing with 20 eq H**_**2**_**O**_**2**_**in the presence and absence of 1 mM Trp.***A*, enzyme alone mixed with 20 eq H_2_O_2_. The spectra depict the formation of intermediate within 11s. *B*, enzyme mixed with 20 eq H_2_O_2_ in the presence of 1 mM Trp. The spectra of ferric ES complex showed marginal changes from 3 ms (*purple*) to 50s (*red*) upon rapid mixing with 20 eq H_2_O_2_. Insets show difference spectra using 3-ms spectra as reference. All spectra were recorded at room temperature.

Heme enzymes are known to activate H_2_O_2_ in the ferric oxidation state to form reactive intermediates such as ferric hydroperoxide and high-valent iron-oxo species such as compound I and II. To gain insights into the potential involvement of a heme-based oxidant in ferric PrnB, we performed stopped-flow absorption spectroscopic experiments. Fe^III^-PsPrnB could readily react with 20 eq H_2_O_2_, showing a decrease in intensity and redshift of the Soret band to 417 nm (Fig. 6*A*). This occurred alongside increases in intensity at 543 and 576 nm and a reduction of the 636-nm band. The intermediate reached its maxima at 11 s with an apparent rate constant of 0.942 s^−1^ when fitting to a single exponential equation (Fig. S9*A*). The intermediate then decayed, and the heme did not return to the resting state, suggesting irreversible heme damage (Fig. S9*B*). Such a phenomenon has been observed in IDO, where the intermediate was assigned as Fe(IV)=O, also known as compound II (40). Next, the reactivity of the intermediate formed in PrnB was examined by mixing the intermediate with Trp. The absence of product in HPLC assays suggests that the intermediate was inactive toward Trp, regardless of whether H_2_O_2_ was added all at once or in aliquots to minimize heme damage (Fig. S10). Hence, this intermediate is not catalytically competent in PrnB. Interestingly, when mixing excess H_2_O_2_ with ferric PrnB precomplexed with 1 mM Trp, the spectra showed almost no change over 50 s (Fig. 6*B*), suggesting that the substrate-bound PrnB was inert to H_2_O_2_. Even with extended incubation times, no significant spectral changes were observed. This contrasts with the IDO reactions, where a compound II-type species formed upon mixing ferric ES complex with H_2_O_2_ (40). The amino group of the substrate blocks the sixth coordination site in PrnB, preventing H_2_O_2_ binding and rendering the enzyme inactive. This could serve as a protective mechanism that prevents the depletion of the primary substrate in a secondary metabolic pathway when the enzyme is in the oxidized state.

The addition of the negatively charged ligand CN^−^ to Fe^III^-PsPrnB immediately formed the CN^−^-bound complex, evident from the Soret shift to 421 nm (Fig. S11*A*). When Trp was subsequentially added, the spectra showed marginal changes, indicating that the heme-bound CN^−^ ligand was unaffected. However, when CN^−^ was added to the ES complex, a noticeable redshift of the Soret band was observed, which eventually transitioned to the CN^−^-bound complex spectrum after 30 min (Fig. S11*B*). This suggests that CN^−^ can slowly displace the amino group. Similar substrate-retarded small-molecule binding has been observed in IDO, where it is attributed to the closed conformation of the ES complex (40). In the case of PrnB, the hindered small-molecule binding may result from both the direct coordination of the substrate's amino group and the closed conformation of the enzyme. Next, we also soaked the 7-Cl-Trp-bound cocrystals in a cyanide-containing mother liquor. This resulted in additional electron density maps in the distal site (Fig. S12). Fitting a substrate molecule in the original amino-ligated conformation or the amino-off conformation could not resolve the additional electron densities, and only the amino-off conformation in addition to the fitting of a cyanide coordination gave the best fitting results. We compared this structure with a previously reported ternary complex structure where the cyanide binding preceded substrate soaking (10). The binding conformation of 7-Cl-Trp aligns with the previously reported structure, while the cyanide is oriented differently. This could result from the reversed binding order of CN- and 7-Cl-Trp. The displacement of the amino group by cyanide in our results suggests that the substrate-coordinated state is likely on pathway.

### Interactions of ferrous PrnB with nitric oxide and oxygen

To assess whether small molecules could replace the amino group in the reduced low-spin ES complex, we used ^•^NO as an oxygen surrogate and spin probe for EPR measurements (Fig. 3*B*). Fe^II^-PsPrnB readily bound ^•^NO, forming a low-spin nitrosyl species detected in the *g* = 2 region. The signal closely resembled those observed in TDO (41). Incubating the ligand-bound complexes with ^•^NO produced similar EPR signals. The EPR spectra of Trp- and TAM-bound nitrosyl complexes displayed similar spectral features, with an additional feature at *g* = 2.07 (Trp) or 2.08 (TAM) (marked by asterisk), compared to the E−^•^NO complex. This variation likely stems from interactions between the amine and heme-bound ^•^NO. In contrast, the IDPA-nitrosyl complex showed a spectrum nearly identical to that of the E−^•^NO complex, suggesting that neither the indole ring nor the carboxylate group interacts closely with the nitrosyl ligand. Cu-EDTA was used as a standard to quantify the nitrosyl signals, and the concentrations and formation yields of the nitrosyl complexes are summarized in Table S2. For the ligand-free sample, the nitrosyl complex formed at 55% relative to the total heme concentration, while the Trp-, TAM-, and IDPA-bound samples showed yields of 43%, 73%, and 62%, respectively. This result aligns with our rR spectroscopic data, where the Trp-bound ferrous enzyme exists in a 6cLS state, while TAM- and IDPA-bound samples are in a 5cHS state. The 6cLS species slightly hinders ^•^NO binding, resulting in a lower yield of the nitrosyl complex, whereas the 5cHS state favors proximal site accessibility, facilitating the nitrosyl complex formation. The higher yield observed in the TAM-bound complex may result from ^•^NO stabilization by the amino group. These EPR results suggest that in the ferrous form, substrate binding slightly inhibits ^•^NO binding.

**Figure 7:**
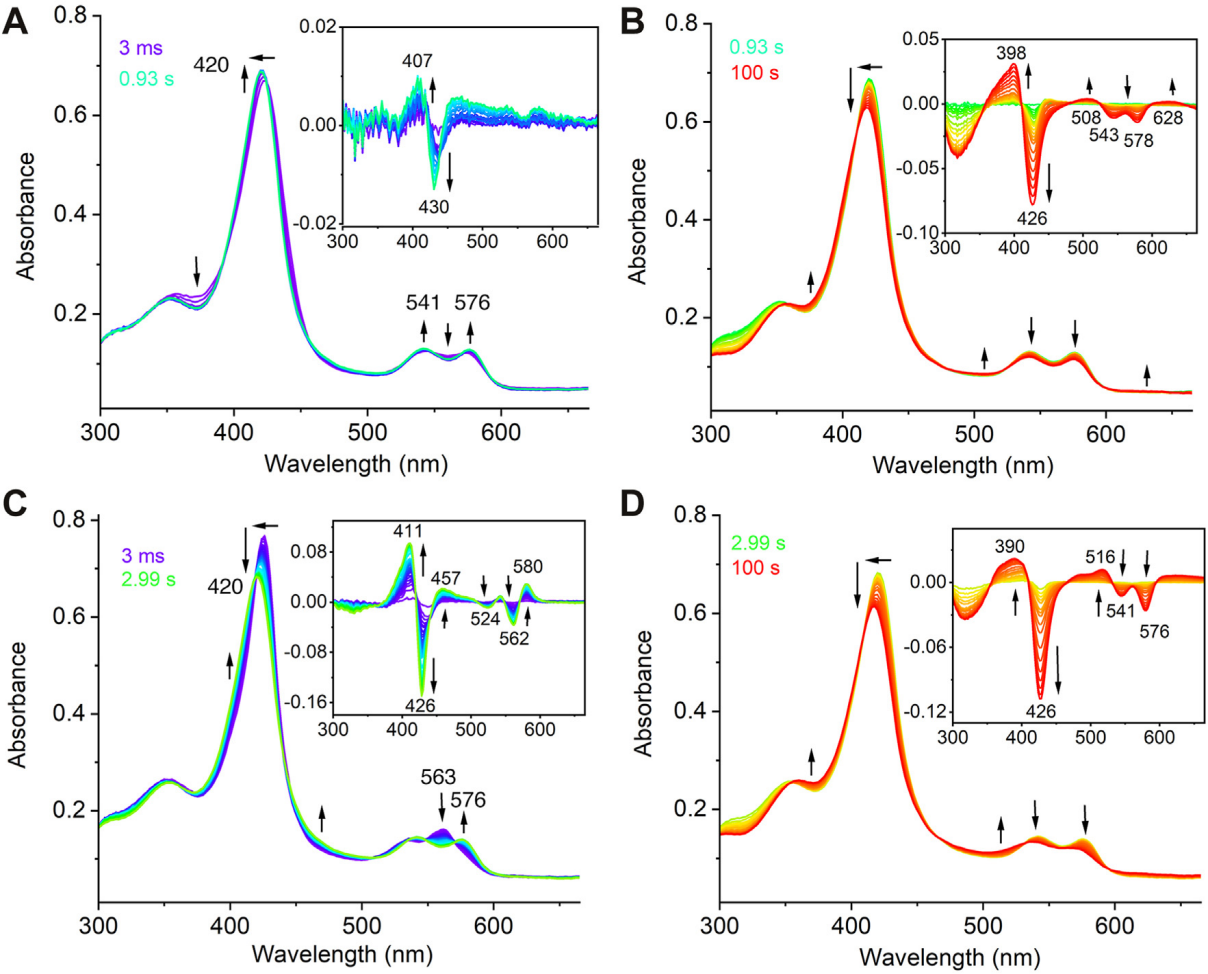
**Stopped-flow photodiode array spectra of ferrous PsPrnB mixing with O**_**2**_**-saturated buffer in the presence and absence of 2 eq Trp.** The spectra of enzyme alone mixed with O_2_-saturated buffer depict (*A*) the formation of an intermediate from 3 ms (*purple*) to 0.93 s (maximum formation, *green*) and (*B*) the slow decay of the intermediate within 100 s (*red*). The spectra of ES complex mixed with O_2_-saturated buffer depict (*C*) the formation of an intermediate from 3 ms (*purple*) to 2.99 s (maximum formation, *green*) and (*D*) the slow decay of the intermediate within 100 s (*red*). Insets show difference spectra. All spectra were recorded at 4 °C.

The binding of ^•^NO to Fe^II^-ES complex prompted stopped-flow spectroscopic investigation into the reactions between PrnB and O_2_. When anaerobic Fe^II^-PsPrnB was mixed with O_2_-saturated buffer at 4 °C, a new species formed rapidly, characterized by a Soret band at 420 nm and Q bands at 541 and 576 nm (Fig. 7*A*). This intermediate slowly decayed after 0.93 s (Fig. 7*B*) and eventually returned to the ferric resting state (Fig. S13*A*). Kinetic analysis of the spectral change at 426 nm over a time course of 100 s revealed a double-exponential fit, with a formation rate constant of 0.691 ± 0.007 s^−1^ and a decay rate constant of 0.0009 ± 0.0001 s^−1^ (Fig. S14*A*). Similar intermediates have been observed in TDO and IDO when the ferrous enzymes react with O_2_ in the absence of the substrate, indicating the formation of a ferrous oxy intermediate (42, 43).

We next mixed the ferrous ES complex with O_2_-saturated buffer to investigate oxygen activation by PsPrnB. The minimum Trp concentration, which is twice the enzyme concentration, was used to fully generate the ES complex. Upon rapidly mixing the ES complex with excess O_2_, an intermediate was observed within the first 2.99 s (Fig. 7*C*). This intermediate is similar to the catalytic ternary complexes formed in TDO and IDO (42, 43, 44, 45). It also resembles the ferrous oxy binary complex formed at 0.93 s in the absence of Trp. The intermediate then slowly decayed to the Fe^III^-ES binary complex, as evidenced by the absence of the 632-nm peak (Figs. 7*D* and S13*B*). This indicates that the substrate was not consumed during the decay of the intermediate, suggesting that the formed ferrous oxy intermediate was catalytically inactive. In TDO and IDO, the ferrous oxy intermediates led to catalytic turnover, as indicated by the increase of the product peak at 321 nm (42, 44). The lack of the product formation in our HPLC analysis further supports the catalytic incompetency of the intermediate (Fig. S10). The kinetic analysis of the spectral change at 426 nm over a time course of 100 s revealed that the process is best fit by a triple-exponential equation (Fig. S14*B*), which is presumably due to the amine dissociation from the ferrous enzyme before O_2_ binding. The enzymatic reactions were subsequently set up under various conditions (see Experimental procedures) that were used for other HDAOs to assess the necessity of common reducing agents and redox proteins. It is unlikely for PrnB to rely on an exclusive redox pair, given that the four *prn* genes alone are sufficient for the production of pyrrolnitrin (4). Despite including ascorbic acid and methylene blue, a standard reducing system in TDO and IDO assays, no product formation was observed. We also tested different redox proteins, putidareductase (PdR)/putidaredoxin (PdX), spinach ferredoxin (SpFdX)/reductase (SpFdR), xanthine oxidase, cytochrome *b*_5_, and cytochrome P450 reductase, but none yielded a detectable product peak. Varying orders of addition, use of both reduced and oxidized enzymes, and use of different oxidants such as O_2_, H_2_O_2_, and *m*-chloroperoxybenzoic acid at different pH levels were also tried, but no reaction product was observed. Further investigation into the cellular activity of PrnB is required to define the enzymatic activity.

Overall, the above experiments suggest that the amine ligation does not hinder the binding and activation of small molecules by ferrous PrnB. The ferrous heme-bound oxy species, an early stage intermediate identified in TDO and IDO, was also formed in PrnB, both in the presence and absence of Trp. However, the formation and decay of the intermediate occurred more rapidly in the presence of Trp (Fig. S14), while the intermediate remained inactive toward the substrate.

## Discussion

The HDAO superfamily comprises an emerging group of His-ligated heme-dependent oxygenases that oxidize aromatic substrates, a reaction typically catalyzed by cysteine-ligated heme enzymes such as cytochrome P450s and peroxygenases. While IDO and TDO have been extensively studied due to their biological significance, the catalytic mechanisms of other HDAO enzymes are still under investigation. In this study, we demonstrate that PrnB exhibits several distinct features compared to other members of the HDAO superfamily and most heme enzymes.

First, PrnB adopts an unusual substrate binding conformation. HDAOs generally bind tyrosine- or tryptophan-based primary substrates in the heme distal pocket, forming a high-spin heme that readily binds and activates small molecules. However, PrnB forms a six-coordinate, low-spin heme upon substrate binding in both ferric and ferrous forms. In UV-visible spectra, the observed redshift of the Soret band in the complexes of 7-Cl-Trp, Trp, and TAM is reminiscent of the Type II binding found in cytochrome P450s, implying that the ligands bind as a sixth ligand to the heme. Further characterization using EPR and rR spectroscopy, X-ray crystallography, and the utilization of substrate analogs confirmed that the type II-like binding in PrnB is due to ligation of the substrate amine. This substrate-binding mode has been observed in some drug-metabolizing P450s, where the type II substrates typically contain a nitrogen atom that provides a lone pair to coordinate with the heme (46, 47). In His-ligated heme systems, the formation of a low-spin species by an endogenous ligand, such as the *bis*-His coordination reported in SfmD (19), is not uncommon. However, nitrogenous substrate coordination to the His-ligated heme to form a HALS species is rare. It may represent a unique strategy used by PrnB to regulate the protonation states of the amino group, thus facilitating the cyclization of the pyrrole ring in its product formation. Further mechanistic investigation is needed to fully understand how this particular substrate coordination contributes to catalysis in PrnB.

PrnB is unique for its interactions with small molecules in the presence of substrate. Enzymes in the HDAO superfamily activate O_2_, H_2_O_2_, or both, to functionalize their aromatic substrates. PrnB is postulated to be an oxygenase but is unique among HDAO enzymes since it produces a non-oxygenated product. This makes it particularly important to understand how PrnB interacts with small-molecule oxidants. TDO, IDO, and MarE are O_2_-dependent enzymes that are active in the ferrous oxidation state, and the oxidized enzymes do not form the expected products when reacting with H_2_O_2_ (20, 32, 39). In contrast, oxidized TyrH and SfmD can readily generate products upon the addition of H_2_O_2_, and the products were also detected when mixing the reduced enzymes with O_2_ in the presence of reducing agents (19, 25). Our stopped-flow experiments show that binding of H_2_O_2_ to the ferric ES complex is inhibited due to amino ligation. However, O_2_ can displace the amino group, and the O_2_ binding process occurs more rapidly in the presence of Trp than in its absence. If the sixth coordination site is involved in oxidant binding, the ferrous oxidation state may be preferred for catalysis. Additionally, since Trp is a primary metabolite and serves as an alternative substrate of PrnB, the formation of inert ferric ES complex may help prevent the depletion of the primary metabolite under oxidized conditions. PrnB is known to catalyze the reaction *in vivo* (3, 10), but once purified, we were unable to replicate this enzymatic activity. This suggests that one or more cellular components may be essential for activating PrnB. Possible missing elements may be small molecules, redox cofactors, partner proteins, or posttranslational modifications that are present in the cellular environment but absent in our *in vitro* assays. We tested several redox agents and proteins commonly active in other heme systems, yet none were successful in restoring activity. The exact nature of these functional components remains unclear, and further investigation into PrnB’s cellular machinery and cofactor requirements will be necessary.

Lastly, PrnB does not rely on the heme-based intermediates that are well-characterized in IDO and TDO. The proposed catalytic mechanisms of IDO and TDO involve stepwise oxygen addition though a Criegee-type rearrangement (Fig. S15) (44, 48, 49). In these enzymes, oxygen addition to the ferrous heme results in the formation of an oxyferrous or ferric superoxo intermediate. These intermediates attack the substrate indole ring to form an alkylperoxo species, which then decays into compound II and a substrate-derived epoxide, with the ferryl species facilitating the second oxygen transfer. The catalytic mechanism of PrnB, as suggested by Naismith *et al.*, is based on standard indole and peroxy chemistry (10, 13). Given the high structural similarity between IDO/TDO and PrnB, some early stage heme-bound intermediates, such as oxyferrous and alkylperoxo species, were thought to be conserved in PrnB catalysis. In the proposed mechanism (10, 13), the oxyferrous species initiates the reaction by electrophilic aromatic substitution to generate an alkylperoxo intermediate, which undergoes heterolytic O-O bond cleavage to form a C3-hydroxylated indole and a ferryl intermediate. Ring rearrangement yields a tricyclic intermediate, which then decomposes to the final product. However, there is currently no experimental evidence to support the mechanism. Our work investigated the catalytic competency of the putative intermediates by transient kinetic study and HPLC activity analysis. The results exclude the possibility of oxygen transfer mediated by these intermediates, suggesting that the PrnB mechanism cannot be simply extrapolated from those of IDO and TDO. Further studies should explore other heme-based oxidants, such as ferric peroxide and compound I, to better elucidate the unique enzymatic catalysis of PrnB. Overall, our findings highlight the unique characteristics of PrnB within the HDAO superfamily and underscore the importance of further investigations to fully understand its catalytic mechanism and biological role.

## Experimental procedures

### Protein expression and purification

The codon-optimized gene of PrnB from *Pseudomonas sp. ADAK18* was cloned into a pET28a-TEV expression vector (GenScript) with a TEV protease-cleavable *N*-terminal His_6_-tag. To improve the folding and solubility of PrnB, *E. coli* BL21(DE3) competent cells (Merck) were transformed with the PrnB plasmid and a pGro7 plasmid (Takara Bio) for the coexpression of GroES and GroEL chaperone proteins. The cells harboring both plasmids were cultivated in 500 ml LB broth containing kanamycin (50 μg/ml), chloramphenicol (25 μg/ml), and arabinose (3 mg/ml). The detailed procedure followed the reported method we used to express heme proteins (2). After overnight incubation (∼16 h), the cells were harvested by centrifugation and stored at −80 °C.

The frozen cells were resuspended in Buffer A (50 mM Tris–HCl and 200 mM NaCl at pH 8.0) containing β-mercaptoethanol (0.1% v/v) and PMSF (0.1 mM). The cells were lysed by sonication and then centrifuged to collect the supernatant. ÄKTA primer plus protein purification system (GE HealthCare) and a HisTrap FF column (Cytiva) were used to isolate PrnB from the supernatant. Buffer A was used for sample loading and initial wash. A gradient of Buffer B (50 mM Tris–HCl, 200 mM NaCl, and 500 mM imidazole at pH 8.0) was applied, and PrnB eluted around 20% Buffer B. All samples and buffers were kept cold during purification. The presence and purity of PrnB in eluted fractions were checked by SDS-PAGE (Fig. S1). The fractions containing pure PrnB were combined and buffer exchanged (Cytiva, HiTrap Desalting column) into a desalting buffer of 100 mM Tris–HCl, 150 mM NaCl, and 5% glycerol at pH 7.5. The resulting PrnB samples were concentrated, aliquoted, and flash-frozen using liquid nitrogen and stored at −80 °C for future use. The concentration of heme-bound PrnB was determined by an extinction coefficient of 140 mM^−1^ cm^−1^ at 406 nm using a pyridine hemochromogen assay (50).

PrnB from *F. bacterium* was expressed and purified following the same protocols, with the only difference being that it did not require a chaperone protein.

### UV-visible and EPR spectroscopic measurements

The PrnB samples subjected to spectroscopic measurements were freshly buffer exchanged (100 mM Tris–HCl, 50 mM NaCl, pH 7.5). Ligand-bound samples were prepared by adding 1 mM 7-Cl-Trp (98%, AChemblock), Trp (99%, BeanTown Chemical), TAM (≥98%, Thermo Fisher Scientific), and IDPA (≥95%, Matrix Scientific). For UV-visible absorption spectral measurements, the samples contained a protein concentration of 10 μM, and all spectra were recorded at room temperature using an Agilent Cary 3500 UV-visible spectrophotometer. The *K*_D_ values were obtained by fitting the titration curves to a hyperbola equation ($\Delta A=\frac{\Delta A_{\max}\times \left[L\right]}{K_{D}+\left[L\right]}$) used for the one-site binding model, where *ΔA* represents the peak-to-trough absorbance difference and [*L*] is the ligand concentration. The reduced samples were prepared by incubating with 2 eq sodium dithionite and measured in a sealed quartz cuvette.

Samples for EPR were prepared using 120 μM protein and flash-frozen in 4 mm quartz EPR tubes. X-band continuous-wave EPR spectra were recorded using a Bruker E560 spectrometer equipped with a cryogen-free 4 K temperature system. For the ferric samples, spectra were taken at various temperatures (10–40 K) and 1 mW microwave power. To prepare nitrosyl complex samples, the protein was reduced by 1 mM sodium dithionite and then exposed to excess ·NO liberated by DEA NONOate (≥98%, Cayman Chemical) for 10 min before being frozen in liquid nitrogen. The entire process was performed in a glove box to prevent oxidation. The spectra of the nitrosyl samples were measured at 30 K and 0.05 mW microwave power. All samples were scanned once using the SHQE high-Q resonator (9.37 GHz frequency) with a field modulation of 100 KHz and an amplitude of 0.6 mT.

### Resonance Raman spectroscopic measurements

Samples for rR spectrometry were prepared with 135 μM protein in NMR tubes and measured at room temperature. The spectra of ferric PrnB and complexes with 1 mM substrates or analogs were obtained using the 405 nm excitation line provided by an OBIS LX 405 nm single frequency diode laser (Coherent). A 405-nm NoiseBlock ASE (amplified spontaneous emission) filter (Ondax) was incorporated to suppress the spontaneous emission from the diode laser. The laser power at the sample was ∼5 mW. The spectra of ferrous PrnB complexes were obtained using 442 nm excitation line from a helium-cadmium laser (Kimmon Koha). The laser power at the sample was ∼10 mW. The scattered beam was collected using 180° backscattering geometry to the incident beam and focused by a cylindrical focusing lens (Nikon). After passing through a 442-nm notch filter (Iridian) or a 405-nm notch filter (Thorlabs), the beam entered an iHR550 spectrometer (HORIBA Scientific) through a 200 μm entrance slit, where it was dispersed by a 1200 grooves/mm grating and then detected by a liquid-nitrogen cooled Symphony charge-coupled device detector (HORIBA Scientific). All samples were measured at room temperature in a spinning NMR tube to avoid local heating and protein degradation. The acquisition time was 2 h for all spectra obtained. It is noted that electronic absorption spectra for each sample was acquired before and after rR measurements and the spectra were identical, indicating no photodegradation of the samples during Raman measurements; ferrous samples remained intact during the entire acquisition period. rR spectra were calibrated with fenchone and processed with Grams A1 software (Thermo Fisher Scientific; https://www.thermofisher.com/order/catalog/product/INF-15000).

### Stopped-flow absorption spectroscopy

Samples for stopped-flow were carried out in absorbance kinetic mode on an Applied Photophysics SX20 stopped-flow system. A buffer containing 100 mM Tris–HCl and 50 mM NaCl at pH 7.5 was used for all mixing. Ferric spectra were obtained by mixing 22 μM protein in the presence or absence of 1 mM Trp with 20 eq H_2_O_2_ at room temperature. Anaerobic ferrous samples were prepared by prereducing the protein with 2 eq sodium dithionite at 4 °C in glovebox (Coy Lab). The reduced enzyme was incubated with 2 eq Trp in anaerobic buffer to produce prominent spectral features at minimum substrate concentrations. The spectra were obtained by mixing 22 μM of the reduced protein samples with O_2_-saturated buffer. The experimental data were analyzed by the ProK software package obtained from Applied Photophysics.

### Activity assessment

Unless otherwise specified, the standard assay was performed in a 200 μl reaction volume containing 50 μM oxidized PrnB and 1 mM substrate (both 7-Cl-Trp and Trp were tested) in a reaction buffer (100 mM Tris–HCl and 150 mM NaCl at pH 7.5). Various oxidants and redox systems were explored under the following conditions. Condition 1: 1 mM H_2_O_2_; condition 2: 1 mM O_2_, tested both in the presence and absence of 5 mM ascorbic acid and 10 μM methylene blue; condition 3: 300 μM xanthine and 50 nM xanthine oxidase, tested both in the presence and absence of O_2_; condition 4: 5 mM *m*-chloroperoxybenzoic acid; condition 5: 50 μM PdR, 200 μM PdX, and 1 mM NADH (97%, Thermo Fisher Scientific); condition 6: 50 μM SpFdR, 200 μM SpFdX, and 1 mM NADPH (98%, AmBeed); condition 7: 100 μM cytochrome P450 reductase and 1 mM NADPH; condition 8: 50 μM prereduced cytochrome *b*_5_. Additionally, prereduced ferrous PrnB and O_2_-saturated buffer were also tested under the above conditions.

All assays were mixed at 300 rpm at room temperature for 45 min. After incubation, the mixtures were filtered using a 10-kDa molecular weight cutoff centrifugal filter (Millipore). A 10-μL portion of the filtrate was injected into an InertSustain C18 column (5 μm particle size, 4.6 × 100 mm, GL Sciences Inc) at a flow rate of 1 ml/min. The samples were analyzed using an Ultimate-3000SD HPLC system (Thermo Fisher Scientific) equipped with a photodiode array detector, recording chromatograms over a wavelength range of 190–800 nm. A 23-min gradient elution was performed using 2.4%–20% acetonitrile with 0.1% formic acid.

### X-ray crystallization

FbPrnB was purified using a HiLoad 16/600 Superdex 75 pg column (Cytiva) with a buffer containing 50 mM Tris–HCl and 50 mM NaCl at pH 7.5. Following purification, the protein was concentrated to 40 mg/ml. Crystallization of FbPrnB in complex with 7-Cl-Trp, Trp, IDPA, and TAM was achieved using a solution composed of 0.1 M Bis-Tris (pH 5.5) and 3.0 M sodium chloride. Crystals were grown using a hanging-drop, vapor-diffusion method by mixing 2.5 μl of the protein solution with 2.0 μl of the crystallization solution and incubating at 12 °C. Red crystals appeared after 2 days and reached optimal size within 1 week. Prior to data collection, crystals were cryoprotected with the crystallization solution supplemented with 20% (v/v) glycerol and flash-frozen in liquid nitrogen.

X-ray diffraction data for the Trp-bound complex were collected at beamline 5.0.3 at Lawrence Berkeley National Laboratory. Data for the 7-Cl-Trp, IDPA, and TAM-bound complexes were collected at beamline 19-ID at the National Synchrotron Light Source II. All datasets were collected at 100 K and processed using HKL2000. Molecular replacement was performed using a previously published PrnB structure (Protein Data Bank entry 2V7I) as the search model. The structures were built and refined using the PHENIX software (https://phenix-online.org/) suite. Ligands were incorporated into the model based on electron densities observed in the distal heme pockets. Detailed crystallographic parameters are provided in Table S3.

## Data availability

The crystal structures of FbPrnB binary and ternary complexes have been deposited in the RCSB Protein Data Bank with the PDB code: 9DFG, 9DFI, 9DFL, 9DFM, and 9EA1.

## Supporting information

This article contains supporting information.

## Conflict of interest

The authors declare that they have no conflicts of interest with the contents of this article.
