# Supplementary material for: Elucidating ligand interactions and small-molecule activation in the pyrrolnitrin biosynthetic enzyme PrnB
Source: J Biol Chem. 2024 Dec 25;301(2):108123. doi: 10.1016/j.jbc.2024.108123 (PMC11791213; doi:10.1016/j.jbc.2024.108123)
Supplement: Supporting Information [file mmc1.docx]

**Elucidating Ligand Interactions and Small-Molecule Activation in the Pyrrolnitrin Biosynthetic Enzyme PrnB**

Bingnan Li^1^, Remigio Usai^1^, Jackson Campbell^1^, Yifan Wang^1, *^

^1^Department of Chemistry, University of Georgia, Athens, GA 30602

^*^Corresponding author; email: [wang.yifan@uga.edu](mailto:wang.yifan@uga.edu); ORCID ID: [0000-0003-0378-2469](https://orcid.org/0000-0003-0378-2469).

**Table of Contents**

[Table S1. Wavelength maxima for various PsPrnB species S2](#_Toc182850405)

[Table S2. Quantitative analysis of heme nitrosyl complexes S3](#_Toc182850406)

[Table S3. X-ray crystallography data collection and refinement statistics S4](#_Toc182850407)

[Fig. S1. Protein sequence alignment of PrnB homologs S6](#_Toc182850408)

[Fig. S2. SDS-PAGE analysis of protein fractions from nickel affinity chromatography S7](#_Toc182850409)

[Fig. S3. Absorption spectra of ferrous PsPrnB and its complex with various ligands S8](#_Toc182850410)

[Fig. S4. X-band continuous wave EPR spectra of ferric PsPrnB S9](#_Toc182850411)

[Fig. S5. EPR analysis of the HALS species in Trp-bound PsPrnB S10](#_Toc182850412)

[Fig. S6. UV-vis and rR spectral characterization of FbPrnB S11](#_Toc182850413)

[Fig. S7. Superposition of ligand binding poses in binary complexes S12](#_Toc182850414)

[Fig. S8. Comparison of active site pockets in Trp-bound binary complexes S13](#_Toc182850415)

[Fig. S9. Kinetic analysis of ferric PrnB mixed with 20 eq H_2_O_2_ S14](#_Toc182850416)

[Fig. S10. HPLC analysis of PrnB activity assay S15](#_Toc182850417)

[Fig. S11. Absorption spectra of PrnB bound with cyanide S16](#_Toc182850418)

[Fig. S12. Crystal structure of FbPrnB in complex with 7-Cl-Trp and cyanide S17](#_Toc182850419)

[Fig. S13. Stopped-flow absorption spectra of ferrous PsPrnB mixed with O_2_-saturated buffer S18](#_Toc182850420)

[Fig. S14. Kinetic analysis of ferrous PsPrnB mixed with O_2_-saturated buffer S19](#_Toc182850421)

[Fig. S15. A general catalytic mechanism for TDO and IDO S20](#_Toc182850422)

[Preparation of redox proteins used in activity assessment S21](#_Toc182850423)

Table S1. Wavelength maxima for various PsPrnB species

|  | Soret band (nm) | Q bands (nm) | CT band (nm) |
| --- | --- | --- | --- |
| Fe^III^-E | 406 | 536; 568 | 636 |
| Fe^II^-E | 426 | 558 |  |
| Fe^III^-E + 7-Cl-Trp | 412 | 535; 563 |  |
| Fe^II^-E + 7-Cl-Trp | 425 | 535; 563 |  |
| Fe^III^-E + Trp | 414 | 537; 563 |  |
| Fe^II^-E + Trp | 425 | 534; 563 |  |
| Fe^III^-E + TAM | 411 | 538; 565 |  |
| Fe^II^-E + TAM | 426 | 561 |  |
| Fe^III^-E + IDPA | 407 | 498; 537; 572 | 624 |
| Fe^II^-E + IDPA | 426 | 560 |  |
| Fe^III^-E + CN^-^ | 421 | 539;568 |  |
| Fe^III^-E + H_2_O_2_ at 11 s | 417 | 543; 576 |  |
| Fe^II^-E + O_2_ at 0.93 s | 420 | 541; 576 |  |
| Fe^II^ ES + O_2_ at 2.99 s | 420 | 541; 576 |  |

Table S2. Quantitative analysis of heme nitrosyl complexes

| Sample | Concentration (μM) | Nitrosyl complex formation yield (%)^a^ |
| --- | --- | --- |
| PsPrnB nitrosyl complex | 40.5 | 55.3 |
| Trp-bound  PsPrnB nitrosyl complex | 31.8 | 43.4 |
| TAM-bound  PsPrnB nitrosyl complex | 53.3 | 72.8 |
| IDPA-bound  PsPrnB nitrosyl complex | 45.3 | 61.9 |

^a^ The formation yield is calculated based on the total heme concentration of 73.2 μM.

Table S3. X-ray crystallography data collection and refinement statistics

| **PrnB complexes** | **7-Cl-Trp bound** | **Trp bound** | **IDPA bound** | **TAM bound** | **7-Cl-Trp and CN^-^ bound** |
| --- | --- | --- | --- | --- | --- |
| PDB code | 9DFG | 9DFI | 9DFL | 9DFM | 9EA1 |
| **Data Collection** | | | | | |
| Wavelength (Å) | 0.97857 | 0.97648 | 0.97857 | 0.97857 | 0.97857 |
| Space group | *C*2 | *C*2 | *C*2 | *C*2 | *C*2 |
| Cell dimensions | | | | |  |
| *a, b, c* (Å) | 142.0, 122.5, 66.7 | 141.1, 122.5, 66.8 | 142.2, 122.6, 66.8 | 140.7, 122.4, 66.5 | 142.5, 122.6, 67.0 |
| ⍺, β, ɣ (˚) | 90.0, 94.6, 90.0 | 90.0, 94.7, 90.0 | 90.0, 94.5, 90.0 | 90.0, 94.6, 90.0 | 90.0, 94.5, 90.0 |
| Resolution (Å) | 50.00−2.42  (2.46−2.42)^a^ | 50.00−2.03  (2.07−2.03) | 50.00-2.50  (2.54-2.50) | 50.00-2.25  (2.29-2.25) | 50.00-2.30  (2.34-2.30) |
| Redundancy | 6.6 (5.5) | 6.7 (5.6) | 6.5 (5.2) | 6.2 (4.4) | 5.9 (3.8) |
| *R*_merge_^b^ (%) | 15.7 (96.8) | 15.0 (90.7) | 23.9 (97.6) | 15.5 (83.5) | 20.7 (99.7) |
| *I*/*σ* | 9.6 (1.3) | 12.4 (1.1) | 8.0 (1.1) | 8.5 (1.2) | 7.8 (1.1) |
| Completeness (%) | 100.0 (99.9) | 98.0 (86.5) | 99.1 (98.2) | 98.9 (89.8) | 99.2(92.7) |
| CC_1/2_, highest resolution shell | 0.63 | 0.63 | 0.60 | 0.65 | 0.54 |
| **Refinement** | | | | | |
| Resolution (Å) | 46.63 −2.42 | 46.50 − 2.02 | 46.75 − 2.51 | 46.11− 2.25 | 46.41− 2.29 |
| No. of reflections | 42,745 | 71,622 | 38,443 | 52,512 | 50,174 |
| *R*_work_^c^/*R*_free_^d^ (%) | 18.09/23.37 | 21.84/26.04 | 20.08/25.78 | 20.63/26.01 | 18.54/23.11 |
| No. atoms/*B*-factors (Å^2^) | | | | | |
| Protein | 5560/41.13 | 5599/32.91 | 5522/31.89 | 5501/33.57 | 5494/35.43 |
| Heme | 86/34.18 | 86/25.08 | 86/23.33 | 86/26.63 | 86/28.43 |
| Ligand | 32/36.54 | 30/28.15 | 28/26.62 | 36/32.96 | 7-Cl-Trp: 32/29.94  CN^-^: 4/28.56 |
| Solvent | 294/44.15 | 568/38.49 | 316/33.45 | 475/38.85 | 503/40.84 |
| Bond lengths (Å) | 0.008 | 0.008 | 0.008 | 0.008 | 0.010 |
| Bond angles (˚) | 0.983 | 0.948 | 1.010 | 0.953 | 1.074 |
| Ramachandran analysis | | | | | |
| Favored (%) | 97.85 | 98.14 | 96.82 | 98.26 | 97.83 |
| Allowed (%) | 2.15 | 1.86 | 3.18 | 1.74 | 2.17 |
| Outlier (%) | 0.00 | 0.00 | 0.00 | 0.00 | 0.00 |

^a^ Numbers in parentheses refer to data in the highest-resolution shell.
^b^ *R*_merge_ = Σ|*I*_h_ − 〈*I*_h_〉|/Σ*I*_h_ , where *I*_h_ is the observed intensity and 〈*I*_h_〉 is the average intensity.
^c^ *R*_work_ = Σ||*F*_o_| − *k*|*F*_c_||/Σ|*F*_o_|.
^d^ *R*_free_ is the same as *R*_obs_ for a selected subset (3-5%) of the reflections that was not included in prior refinement calculations.


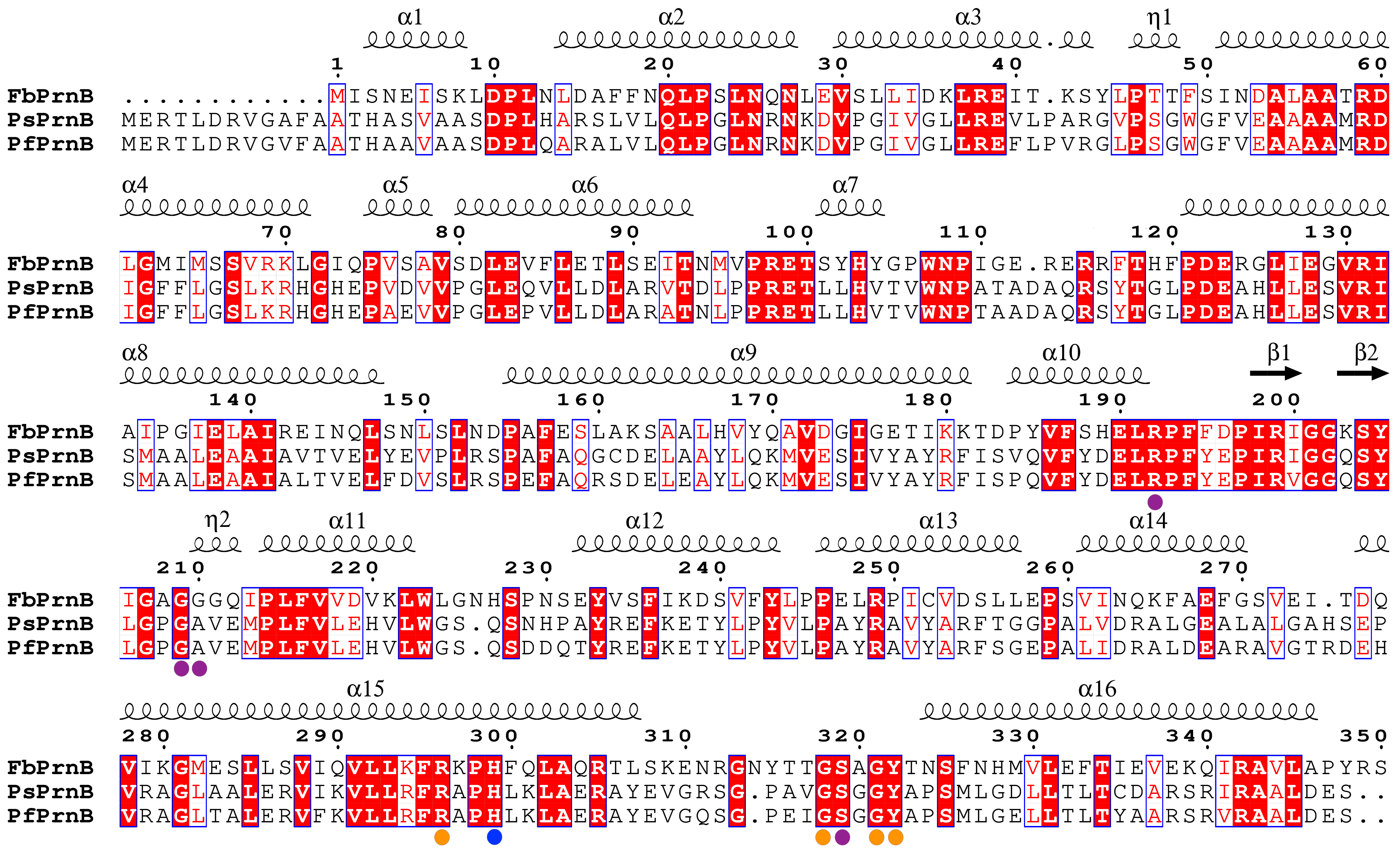


Fig. S1. Protein sequence alignment of PrnB homologs

Selected PrnBs are from *Flavobacteriales* bacterium (FbPrnB, MCB9204939), *Pseudomonas* sp. ADAK18 (PsPrnB, WP_169354555), and *Pseudomonas fluorescens* (PfPrnB, AAB97505). White characters on a red background in blue boxes represent residues of strict identity, while red characters in blue boxes represent residues of similarity. The secondary structure elements are defined by FbPrnB. The heme axial histidine ligand is highlighted by a blue circle; orange and purple circles highlight residues involved in stabilizing the heme and ligands (substrates and small molecules), respectively. ****

Fig. S2. SDS-PAGE analysis of protein fractions from nickel affinity chromatography

The fractions of PsPrnB (left) FbPrnB (right) were eluted with 20% and 10% buffer B, respectively. The recombinant PrnB proteins have a molecular weight of 39 kDa.
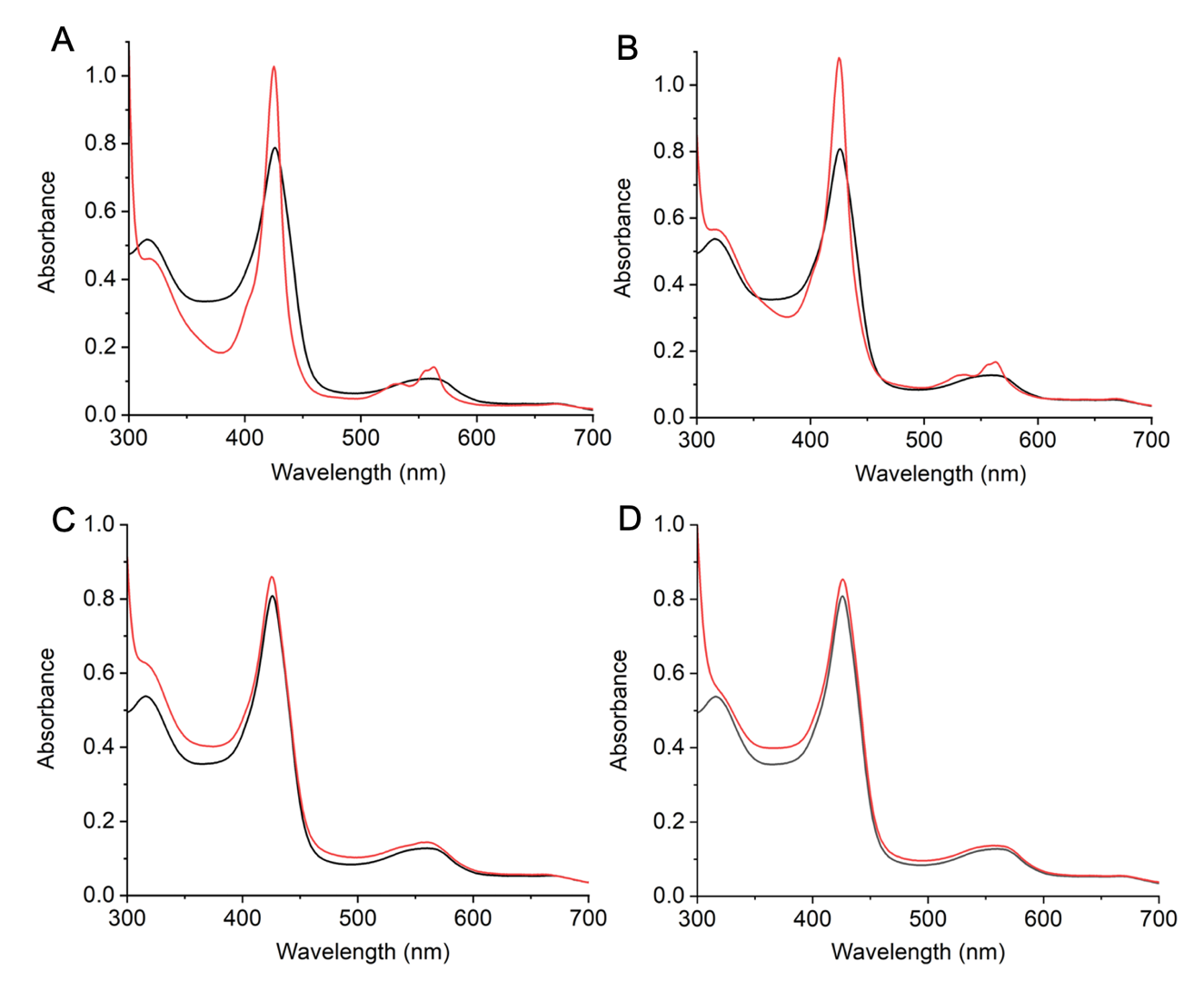


Fig. S3. Absorption spectra of ferrous PsPrnB and its complex with various ligands

Ferrous PsPrnB bound with (**A**) 1 mM 7-Cl-Trp, (**B**) 1 mM Trp, (**C**) 1 mM TAM, and (**D**) 1 mM IDPA. Enzyme alone (black) and enzyme bound with ligands (red).


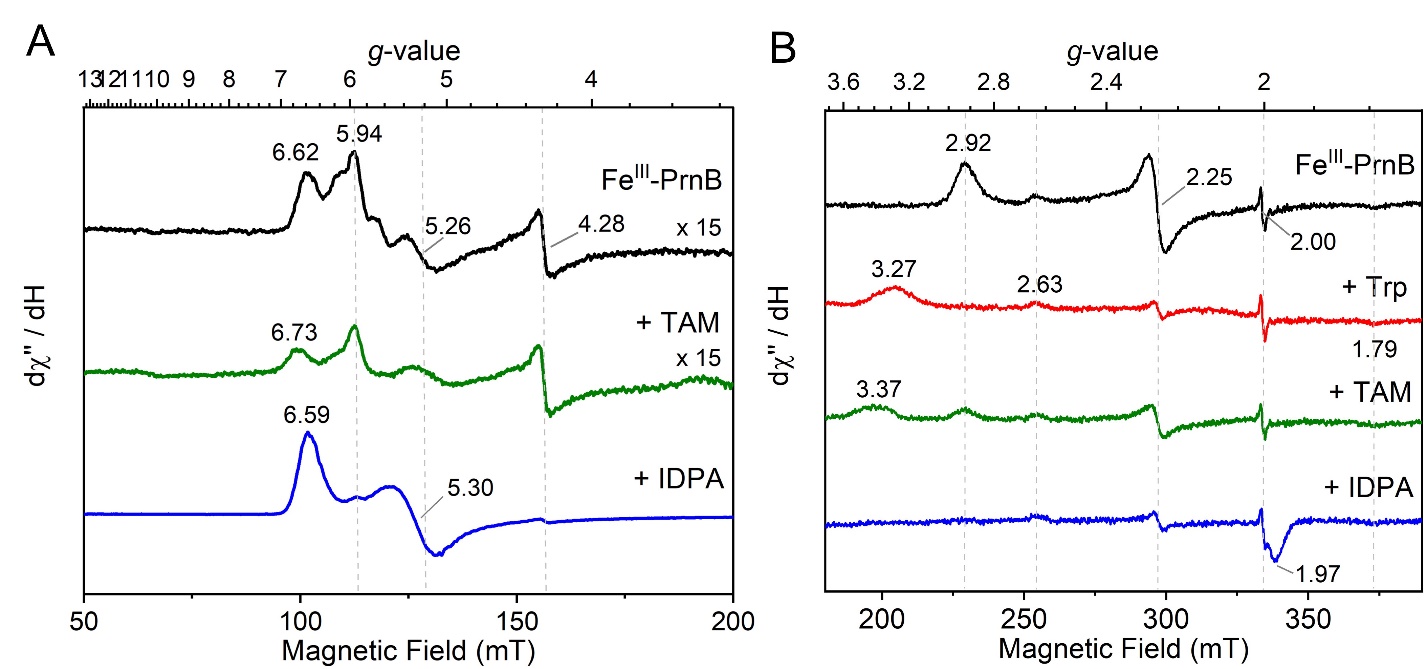


Fig. S4. X-band continuous wave EPR spectra of ferric PsPrnB

(**A**) The high-spin region of ferric PsPrnB and ligand-bound complexes. From top to bottom: ferric PsPrnB enzyme alone (black), with 1 mM TAM (green), and with 1 mM IDPA (blue). The spectra of ferric PrnB and TAM-bound PrnB were magnified by 15-folds. Trp does not have any detectable high-spin signal. The spectra were collected at 10 K and 1.0 mW microwave power. (**B**) The low-spin region of ferric PrnB and ligand-bound complexes. From top to bottom: ferric enzyme alone (black), with 1 mM Trp (red), with 1 mM TAM (green), and with 1 mM IDPA (blue). The spectra were collected at 30 K and 1.0 mW microwave power.


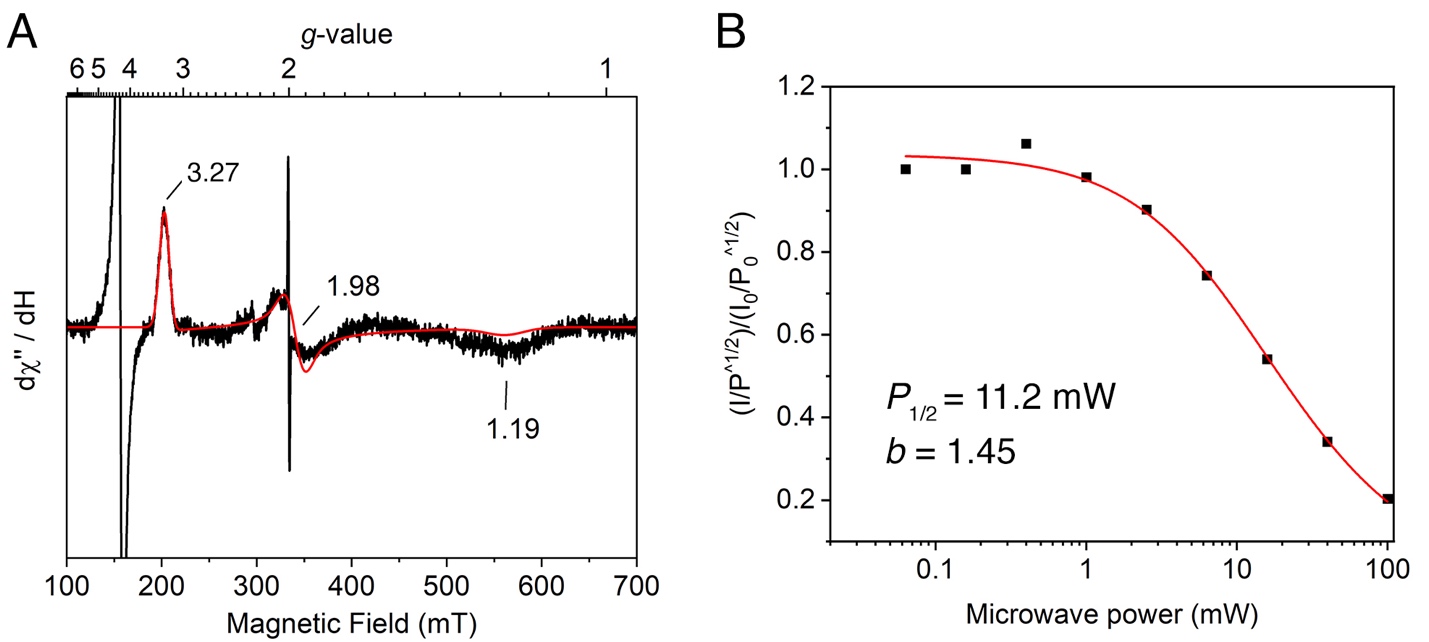


Fig. S5. EPR analysis of the HALS species in Trp-bound PsPrnB

(**A**) EPR spectrum of 0.21 mM PrnB in complex with 2 mM Trp, measured at 10 K and 1 mW. The red trace represents a simulated spectrum generated using the software Spin. The simulated *g*-values for the HALS species are 3.27, 1.98, and 1.19. Signals from the adventitious iron (*g* = 4.28) and the radical-like species (*g* = 2.00) are not simulated. (**B**) Microwave power saturation profile of the HALS signal recorded at *g*_max_ at 3.27 at 10 K. The red trace represents a fitting curve using the equation of $\frac{I/\sqrt{P}}{I_{o}/\sqrt{P_{o}}} = \frac{A}{{(1+\frac{P}{P_{1/2}})}^{b/2}}$, from which the *P*_1/2_ and *b* values are determined.

The power saturation experiments were conducted on a Bruker EMX-Plus EPR spectrometer equipped with a ColdEdge Stinger closed-cycle helium flow system. Measurements were performed at various microwave powers, and all spectra were recorded with four averaged scans.


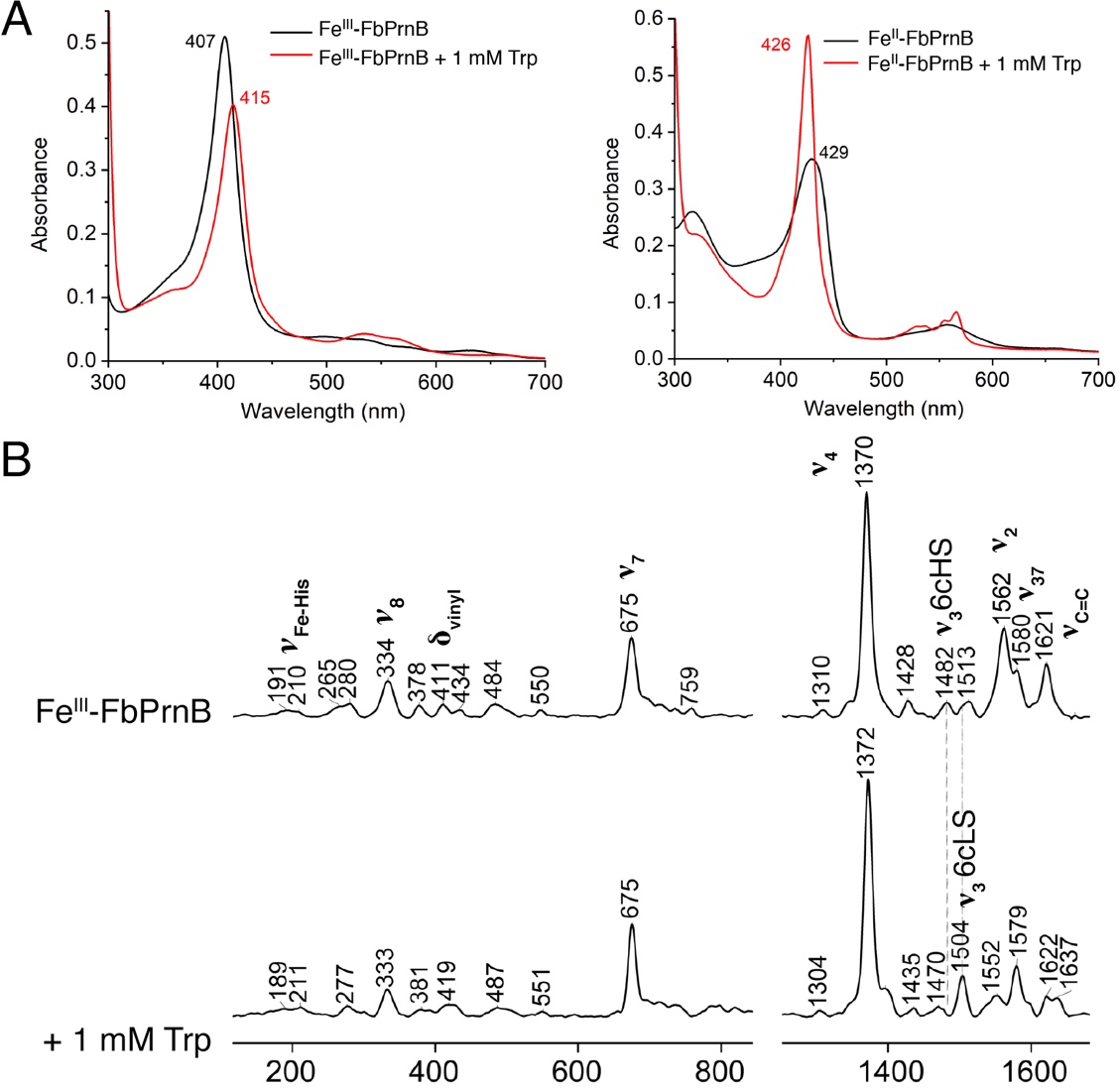


Fig. S6. UV-vis and rR spectral characterization of FbPrnB

(**A**) UV-vis spectra of FbPrnB in the reduced and oxidized forms in the presence and absence of 1 mM Trp. (**B**) rR spectra of FbPrnB in the presence and absence of 1 mM Trp.


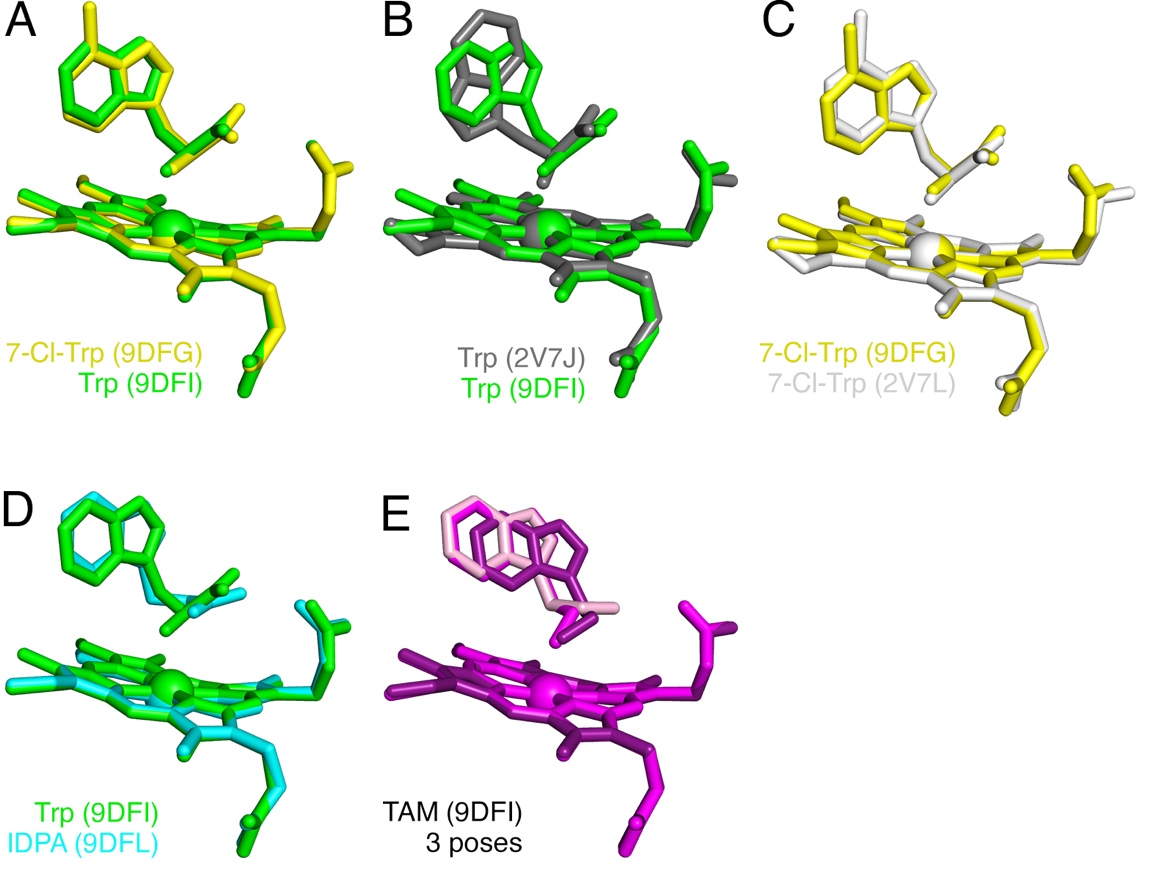


Fig. S7. Superposition of ligand binding poses in binary complexes

(**A**) Binding poses of 7-Cl-Trp (yellow) and Trp (green) from this work; (**B**) Binding poses of Trp from this work (green) and from PDB entry 2V7J (gray); (**C**) Binding poses of 7-Cl-Trp from this work (yellow) and from PDB entry 2V7L (white); (**D**) Binding poses of Trp (green) and IDPA (cyan) from this work; (**E**) Three different binding poses of TAM in Chain A and Chain B from this work.


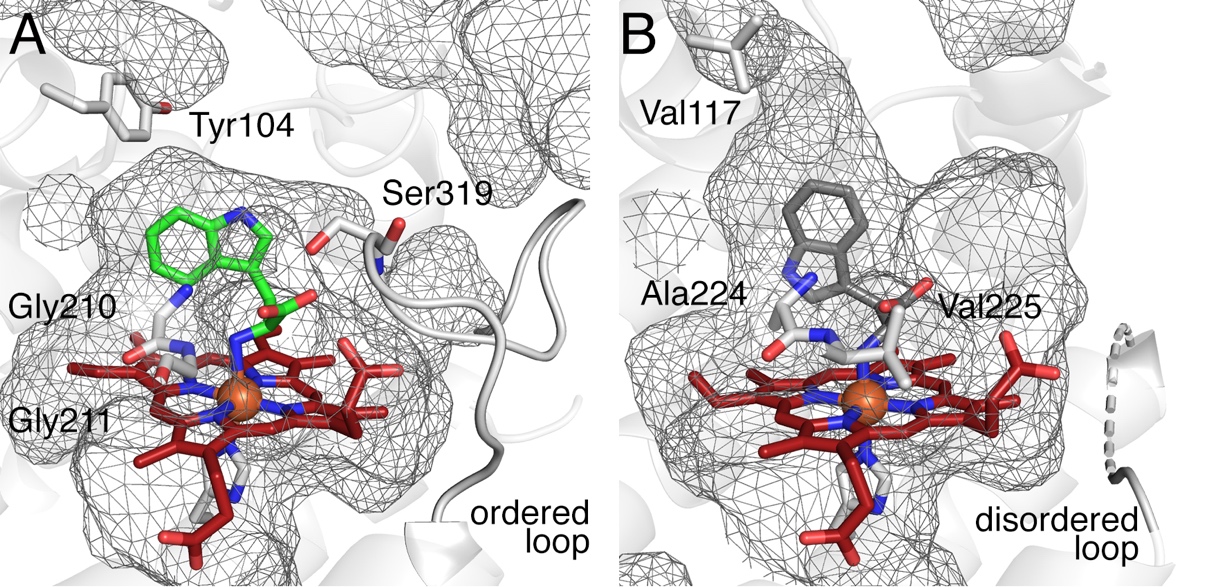


Fig. S8. Comparison of active site pockets in Trp-bound binary complexes

(**A**) Structure of FbPrnB (PDB entry: 9DFI) and (**B**) structure of PfPrnB (PDB entry: 2V7J). Pockets are shown in gray meshes.


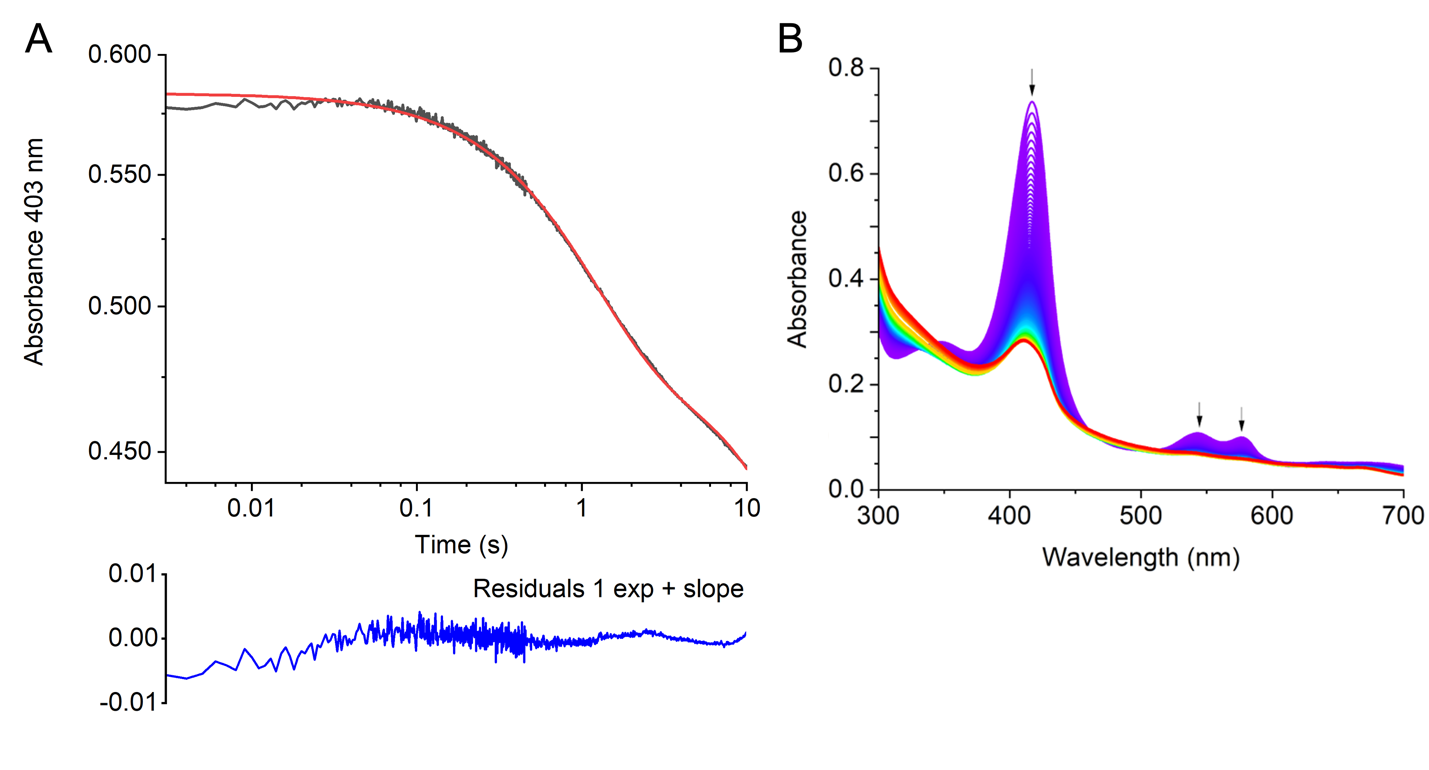


Fig. S9. Kinetic analysis of ferric PrnB mixed with 20 eq H_2_O_2_

(**A**) The time-resolved absorbance change at 403 nm obtained from a stopped-flow spectrometer was fit by a single exponential equation with apparent rate constant of *k* = 0.942 ± 0.005. Stopped-flow data, single exponential fitting, and fitting residuals are shown in black, red, and blue traces, respectively. (**B**) UV-vis spectra of the decay of the intermediate in 100 min at room temperature. Heme was damaged in the presence of excess H_2_O_2_.


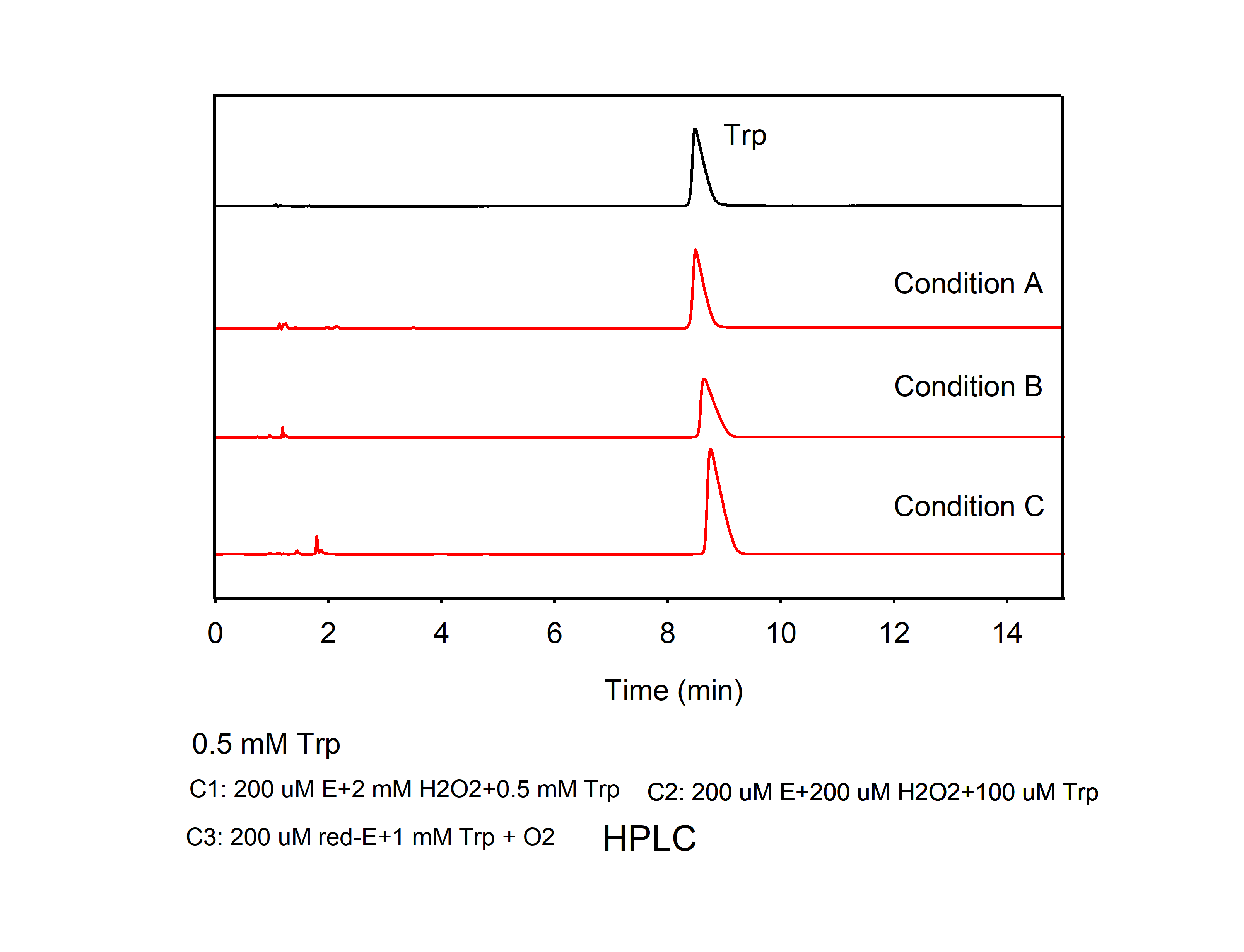


Fig. S10. HPLC analysis of PrnB activity assay

The traces from top to bottom are HPLC analysis of 0.5 mM Trp standard, Condition A: 200 μM ferric PrnB first mixed with 2 mM H_2_O_2_ then with 0.5 mM Trp, Condition B: 200 μM ferric PrnB mixed with 200 μM H_2_O_2_ and 100 μM Trp (added in ten aliquots of 20 uM and 10 uM respectively, in order to avoid heme degradation and substrate inhibition), and Condition C: 200 μM chemically reduced ferrous PrnB mixed with 1 mM Trp and O_2_-saturated buffer. The chromatograms are shown at 280 nm. A 15-min isocratic elution method was implemented using 2.4-10% acetonitrile and 0.1% formic acid.


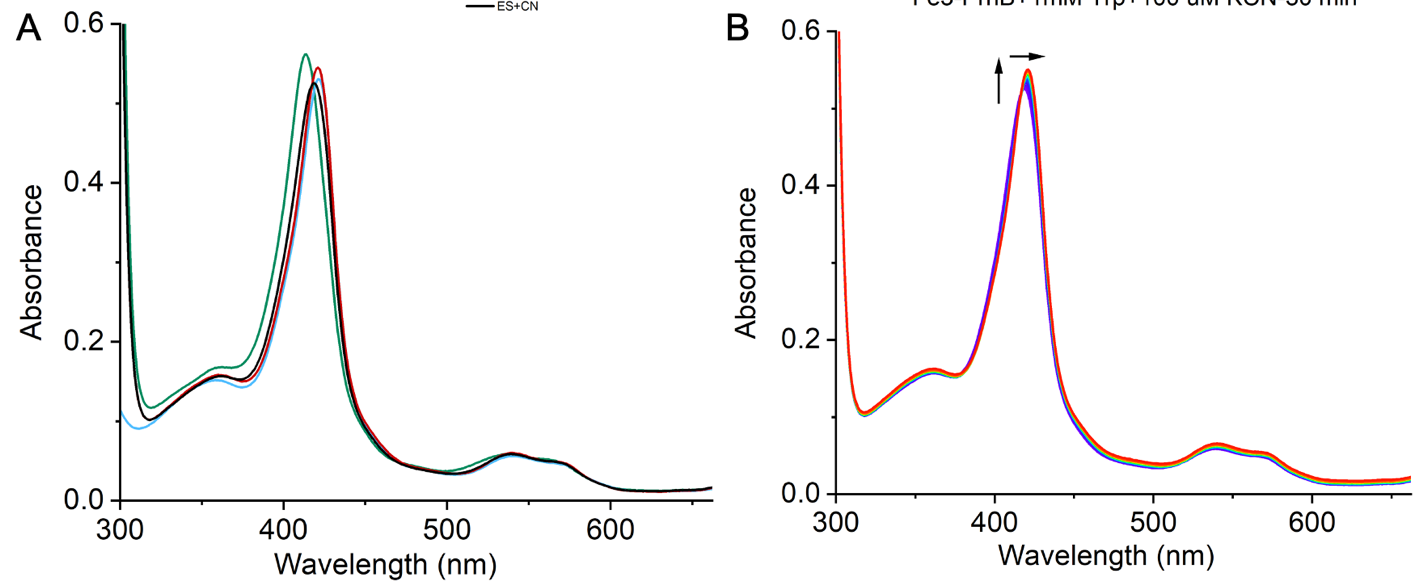


Fig. S11. Absorption spectra of PrnB bound with cyanide

(**A**) Spectra of E-Trp complex (green), E-Trp complex with 10 eq CN^-^ recorded immediately (black), E-CN^-^ complex (blue), and E-CN^-^ complex with Trp (red). (**B**) The spectral changes of E-Trp complex with 10 eq CN^-^ over 30 min. The results show CN^-^ could compete with the substrate amine group, but the binding process is not as rapid as that of enzyme alone.

The PrnB samples were prepared in the buffer (100 mM Tris, 50 mM NaCl, pH 7.51). The samples contained 10 μM protein, 1 mM Trp, and 10 eq KCN (98.5%, Fisher Chemical). All spectra were recorded at room temperature using an Agilent Cary 3500 UV-vis spectrophotometer.


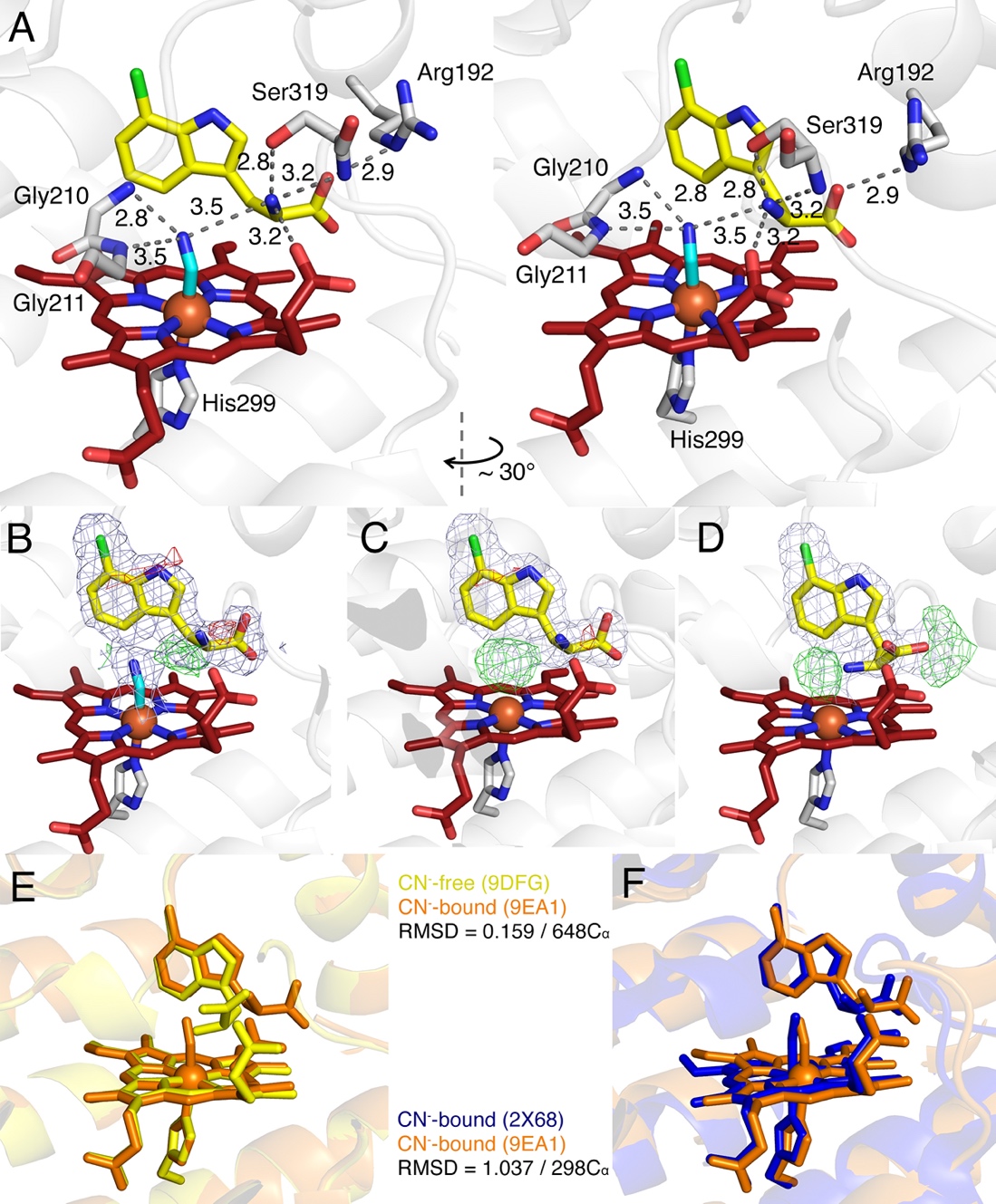


Fig. S12. Crystal structure of FbPrnB in complex with 7-Cl-Trp and cyanide

The structure was obtained from a 7-Cl-Trp-bound co-crystal after soaking in 5 mM KCN for 0.5 hr. The distances (Å) between atoms are represented by gray dashed lines. (**A**) Interactions between the protein and the ligands. (**B**) Electron density maps after fitting CN^-^ and 7-Cl-Trp. (**C**) Electron density maps after fitting 7-Cl-Trp in the amino-off conformation. (**D**) Electron density maps after fitting 7-Cl-Trp in the amino-on conformation. The 2*F*_o_–*F*_c_ difference maps (light blue) for the ligands are contoured at 1.0 σ. The *F*_o_–*F*_c_ difference maps (green and red) for the ligands are contoured at ±3.0 σ. (E) Structural comparison of the CN^-^-free (yellow, PDB entry: 9DFG) and CN^-^-bound (orange, PDB entry: 9DFG) complexes from this study. (F) Structural comparison of the CN^-^-bound 7-Cl-Trp complexes from the prior study (blue, PDB entry: 2X68) and this study (orange, PDB entry: 9EA1).
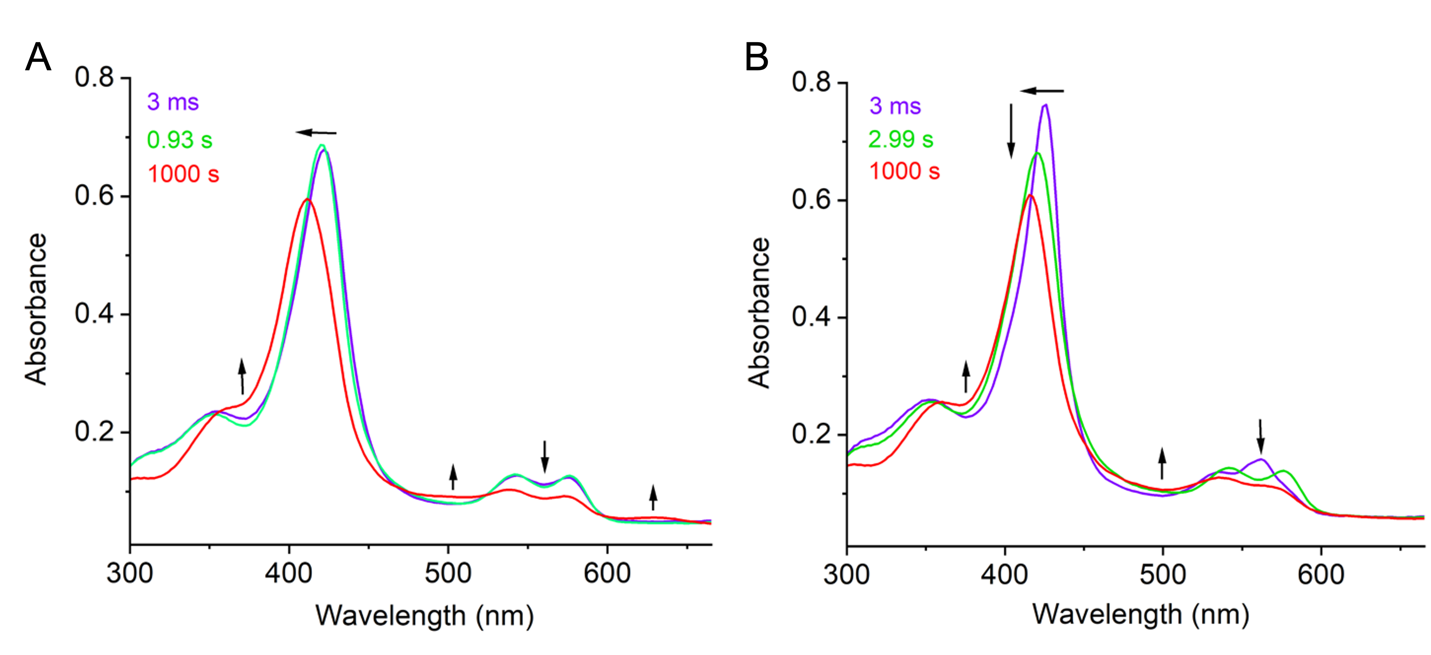


Fig. S13. Stopped-flow absorption spectra of ferrous PsPrnB mixed with O_2_-saturated buffer

(**A**) Spectra obtained at 3 ms (reduced enzyme), 0.93 s (ferrous oxy), and 1000 s (ferric enzyme) in the absence of Trp at 4 ℃. The enzyme returned to the ferric resting state after the reaction. (**B**) Spectra obtained at 3 ms (reduced ES complex), 2.99 s (ferrous oxy), and 1000 s (ferric ES complex) in the absence of Trp at 4 ℃.


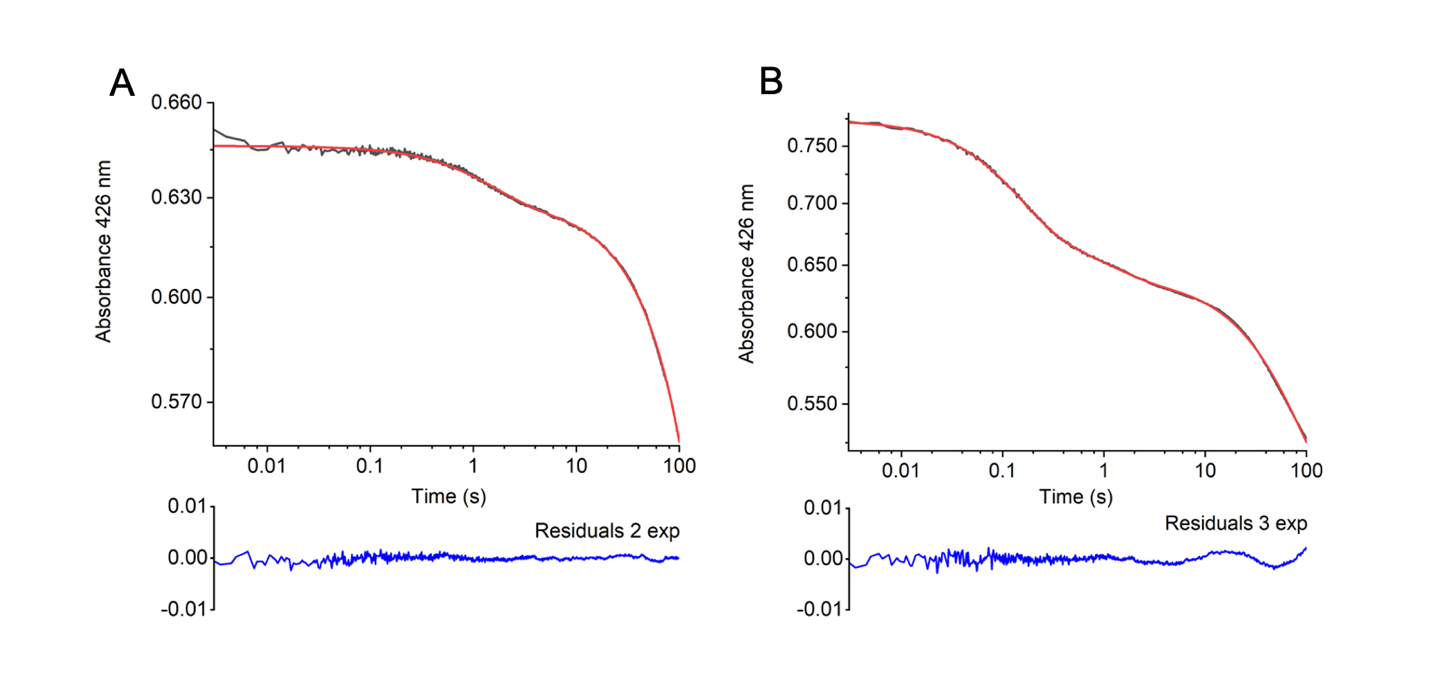


Fig. S14. Kinetic analysis of ferrous PsPrnB mixed with O_2_-saturated buffer

(**A**) Enzyme alone mixed with O_2_-saturated buffer by a stopped-flow spectrometer at 4 ^o^C. The time-resolved absorbance change at 426 nm was fit by a double exponential equation with apparent rate constant of *k*_1_ = 0.691 ± 0.007 and *k*_2_ = 0.0009 ± 0.0001. (**B**) ES complex (prepared with [E]:[S] = 1:2) mixed with O_2_-saturated buffer by a stopped-flow spectrometer at 4 ^o^C. The time-resolved absorbance change at 426 nm was fit by a triple exponential equation with apparent rate constants of *k*_1_ = 7.36 ± 0.05, *k*_2_ = 0.94 ± 0.01, and *k*_3_ = 0.0122 ± 0.0001. Stopped-flow data, fitting, and fitting residuals are shown in black, red, and blue traces, respectively.


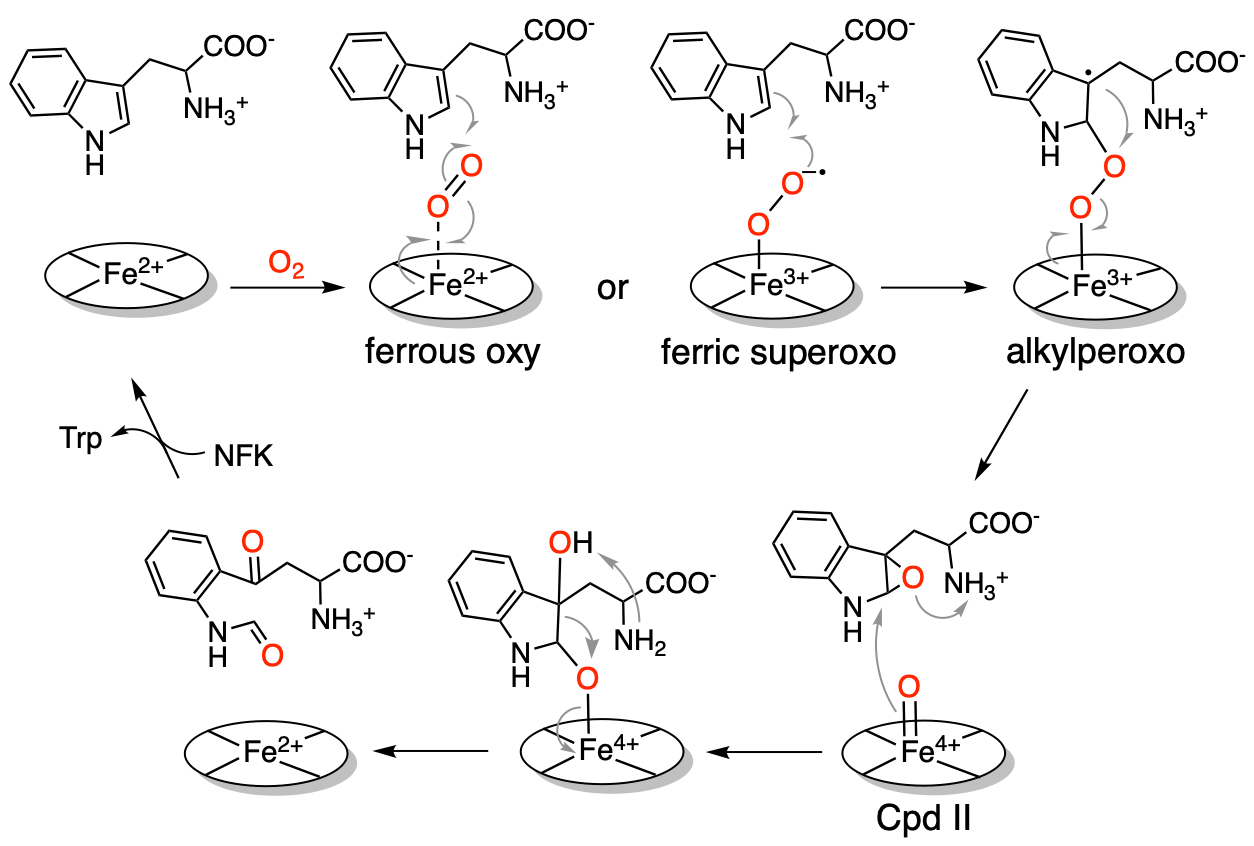


Fig. S15. A general catalytic mechanism for TDO and IDO

Preparation of redox proteins used in activity assessment

The PdX (#85083) and PdR (#85084) plasmids were obtained from Addgene. The genes for CPR, Cyt *b*_5_, SpFdX, and SpFdR were cloned into a pET28a expression vector (Twist Bioscience). The constructs were designed following previously reported methods.[^1-4^](#_ENREF_1) Expression and purification were adapted from the established protocols.[^1-5^](#_ENREF_1)

All plasmids were transformed into *E. coli* BL21 (DE3) cells, which were grown in LB medium supplemented with kanamycin (50 μg/mL) at 37 °C until the OD_600_ reached 0.6. The temperature was then reduced to 20 °C, and IPTG (0.2 mM for PdX, PdR, SpFdX and SpFdR; 10 μM for Cyt *b*_5_; 1.0 mM for CPR) was added to induce protein expression. For the cell cultures of PdX and SpFdX, ferrous ammonium sulfate (20 μg/ml) was also added. For the cell cultures of Cyt *b*_5_, ferrous ammonium sulfate (20 μg/ml) and δ-aminolevulinic acid (40 μg/ml) were also added. The culture was then grown for an additional 16 hours at 20 °C. Cells were harvested by centrifugation and resuspended in Buffer A containing 0.1 mM phenylmethylsulfonyl fluoride. Proteins were isolated using HisTrap FF columns (Cytiva) with a gradient of Buffer B. After purification, the proteins were buffer-exchanged into 100 mM Tris-HCl, 150 mM NaCl, and 5% glycerol at pH 7.5. SDS-PAGE and activity assays confirmed that all proteins were purified in their soluble and active forms. The proteins were then concentrated, aliquoted, flash-frozen with liquid nitrogen, and stored at -80 °C for future use.

1. Binda, C., Coda, A., Aliverti, A., Zanetti, G., and Mattevi, A. (1998) Structure of the mutant E92K of [2Fe-2S] ferredoxin I from *Spinacia oleracea* at 1.7 Å resolution *Acta Crystallogr. D Biol. Crystallogr.* **54**, 1353-1358

2. Bruns, C. M., and Karplus, P. A. (1995) Refined crystal structure of spinach ferredoxin reductase at 1.7 Å resolution: oxidized, reduced and 2'-phospho-5'-AMP bound states *J. Mol. Biol.* **247**, 125-145

3. Beck von Bodman, S., Schuler, M. A., Jollie, D. R., and Sligar, S. G. (1986) Synthesis, bacterial expression, and mutagenesis of the gene coding for mammalian cytochrome *b*_5_ *Proc. Natl. Acad. Sci. U. S. A.* **83**, 9443-9447

4. Marohnic, C. C., Panda, S. P., Martasek, P., and Masters, B. S. (2006) Diminished FAD binding in the Y459H and V492E Antley-Bixler syndrome mutants of human cytochrome P450 reductase *J. Biol. Chem.* **281**, 35975-35982

5. Tan, C. Y., Hirakawa, H., Suzuki, R., Haga, T., Iwata, F., and Nagamune, T. (2016) Immobilization of a bacterial cytochrome P450 monooxygenase system on a solid support *Angew. Chem. Int. Ed. Engl.* **55**, 15002-15006
